# Boosting Lean Electrolyte Lithium–Sulfur Battery Performance with Transition Metals: A Comprehensive Review

**DOI:** 10.1007/s40820-023-01137-y

**Published:** 2023-06-29

**Authors:** Hui Pan, Zhibin Cheng, Zhenyu Zhou, Sijie Xie, Wei Zhang, Ning Han, Wei Guo, Jan Fransaer, Jiangshui Luo, Andreu Cabot, Michael Wübbenhorst

**Affiliations:** 1 Laboratory for Soft Matter and Biophysics, Faculty of Science, KU Leuven, 3001 Leuven, Belgium; 2 Fujian Key Laboratory of Polymer Materials, College of Chemistry and Materials Science, Fujian Normal University, Fuzhou, 350007 People’s Republic of China; 3 Department of Materials Engineering, Faculty of Science Engineering, KU Leuven, 3001 Leuven, Belgium; 4 Lab of Electrolytes and Phase Change Materials, College of Materials Science and Engineering, Sichuan University, Chengdu, 610065 People’s Republic of China; 5 Advanced Materials Department, Catalonia Institute for Energy Research (IREC), Sant Adria del Besos, 08930 Barcelona, Spain

**Keywords:** Transition metals, Lean electrolyte, Sulfur reduction reactions, Li–S batteries

## Abstract

**Abstract:**

Lithium–sulfur (Li–S) batteries have received widespread attention, and lean electrolyte Li–S batteries have attracted additional interest because of their higher energy densities. This review systematically analyzes the effect of the electrolyte-to-sulfur (E/S) ratios on battery energy density and the challenges for sulfur reduction reactions (SRR) under lean electrolyte conditions. Accordingly, we review the use of various polar transition metal sulfur hosts as corresponding solutions to facilitate SRR kinetics at low E/S ratios (< 10 µL mg^−1^), and the strengths and limitations of different transition metal compounds are presented and discussed from a fundamental perspective. Subsequently, three promising strategies for sulfur hosts that act as anchors and catalysts are proposed to boost lean electrolyte Li–S battery performance. Finally, an outlook is provided to guide future research on high energy density Li–S batteries.

**Graphical Abstract:**

## Highlights

- This review systematically analyzes the effect of the electrolyte-to-sulfur ratios on battery energy density and the challenges for sulfur reduction reactions under lean electrolyte conditions.
- The strengths and limitations of different transition metal compounds are systematically presented and discussed from a fundamental perspective.
- Three promising strategies for sulfur hosts that act as anchors and catalysts are proposed to boost lean electrolyte Li–S battery performance.

## Graphical abstract

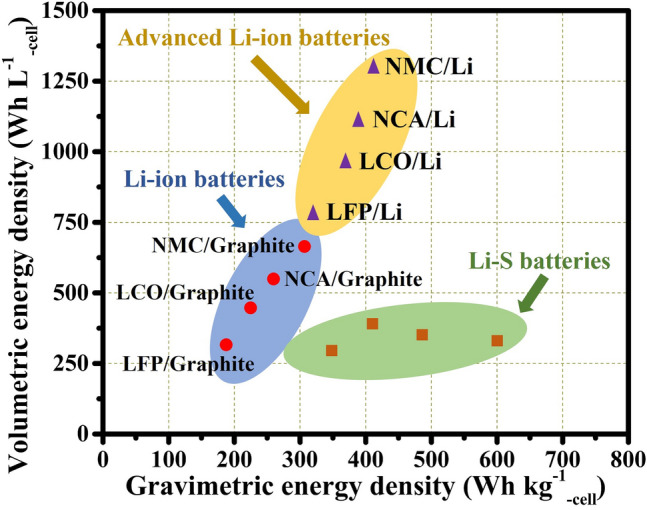

## Introduction

The lithium-sulfur (Li–S) battery is based on a conversion-type cathode where the electrochemical redox reaction between active sulfur (S_8_) and lithium sulfide (S_8_ + 16Li^+^ + 16e^−^ ⇌ 8Li_2_S) takes place [1–3]. While sulfur is very abundant and inexpensive, sulfur cathodes provide much higher theoretical specific capacities (1675 mAh g^−1^) than those of intercalation-type lithium-ion (Li-ion) batteries (e.g., LiFePO_4_-170 mAh g^−1^; LiCoO_2_-274 mAh g^−1^; LiMn_2_O_4_-148 mAh g^−1^) [4–7]. Thus, Li–S batteries are particularly appealing next-generation rechargeable energy storage devices owing to their potential for low cost and high theoretical energy density (2600 Wh kg^−1^) [4, 8–10].

**Fig. 1:**
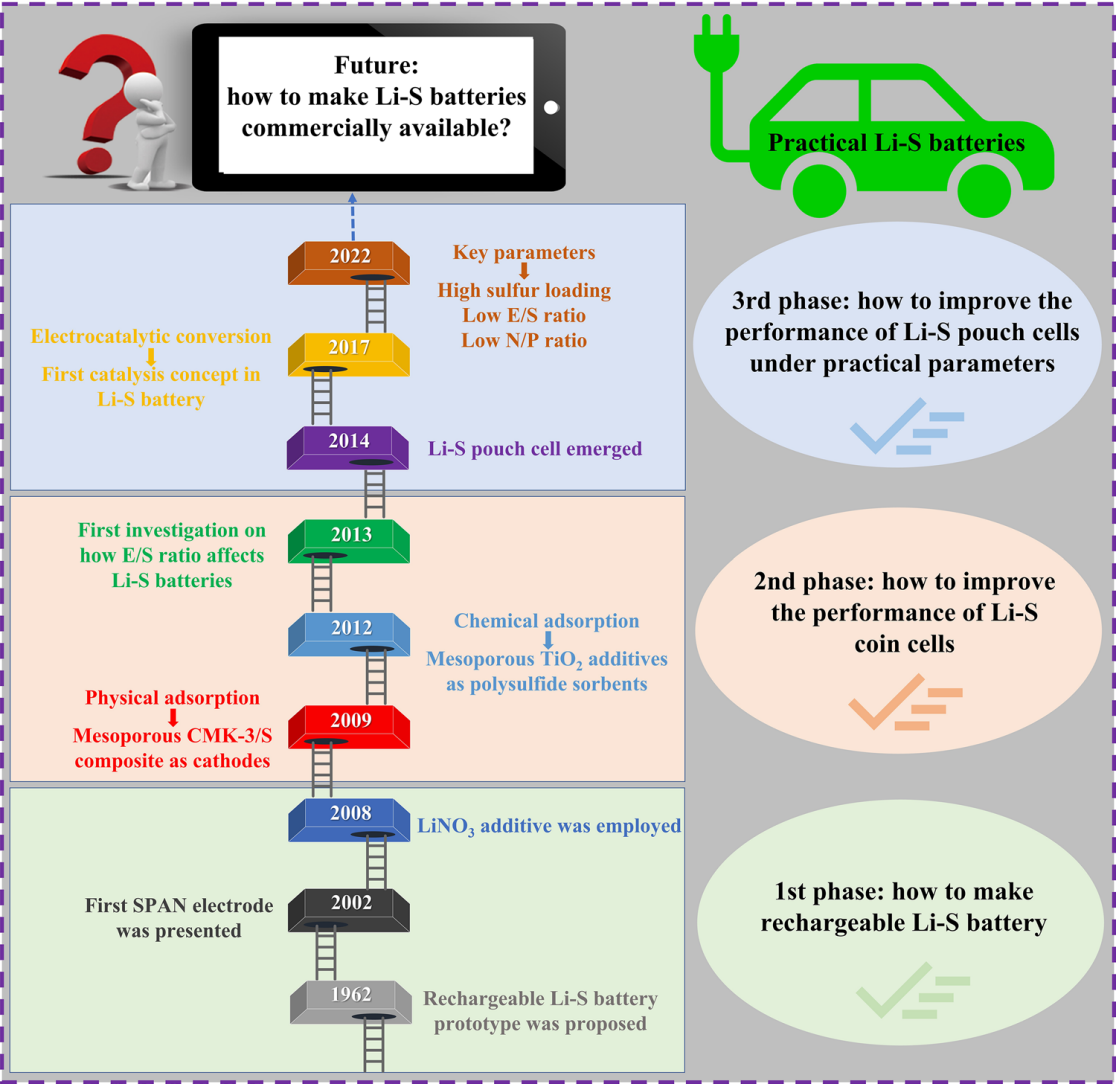
A brief timeline and representative events in the development of Li–S batteries. The development process can be divided into four stages, mainly covering (i) 1962–2008: how to make Li–S battery cycle; (ii) 2009–2013: how to improve the performance of Li–S coin cells; and (iii) 2014-present: how to improve the performance of Li–S pouch cells under practical parameters; (iv) future: how to make Li–S batteries commercially available. Inserted represented works: 1962 [50], 2002 [22]. Copyright 2002, Wiley-VCH. 2008 [23], 2009 [24]. Copyright 2009, Springer Nature. 2012 [51]. Copyright 2012, American Chemical Society. 2013 [26], 2014 [27]. Copyright 2014, Elsevier. 2017 [37]. Copyright 2017, National Academy of Sciences. 2022 [48]. Copyright 2022, Wiley-VCH

Li–S batteries emerged in 1962 when Herbert and Ulam first proposed the concept of employing sulfur as a cathode [11–15]. Looking back over the past 60 years, the development process of Li–S batteries can be divided into three stages (Fig. 1). The first phase focused on how to make rechargeable Li–S batteries (1962–2008). During this development process, conventional organic electrolytes (e.g., propylene carbonate, ethylene carbonate, dimethyl carbonate, dimethyl sulfoxide) [16–18], dioxolane-based organic electrolytes (e.g., 1.3-dioxolane) [19] and polymer electrolytes (e.g., polyethylene oxide) [20, 21] were studied. In 2002, Wang et al. first proposed a sulfur/conductive polymer (sulfur@polyaniline) composite as cathode material for rechargeable batteries with high performance [22]. In 2008, Mikhaylik found that lithium nitrate (LiNO_3_) was an effective electrolyte additive to inhibit the shuttle effect and boost the Coulombic efficiency of Li–S cells [23]. However, at the end of this first phase, the reversibility of the Li–S batteries was still very low. The second phase primarily focused on improving the performance of Li–S coin cells (2009–2013). In 2009, Nazar’s group achieved more than 20 stable cycles of Li–S cells by using polymer-modified mesoporous carbon (CMK-3) as a sulfur host [24], which sparked a renaissance of the Li–S battery. Following that, various porous carbon and polar materials were developed to mitigate the dissolution of lithium polysulfides (LiPS) through physical confinement or chemical adsorption [1, 25]. In 2013, Xiao et al. explored the effect of the electrolyte/sulfur (E/S) ratio on Li–S batteries [26] evidencing the great challenge that is moving toward high-density lean electrolyte Li–S batteries [27, 28]. In this second phase, multiple strategies such as advanced sulfur hosts [24, 29], anode protection [30, 31] and electrolyte optimization [32, 33], were employed to develop Li–S coin cells reaching lifespans over 1000 cycles [34]. However, to demonstrate practical energy densities above 400 Wh kg^−1^, Li–S pouch cells with practical parameters had to be developed. The third phase (2014–now) focuses on improving the performance of Li–S pouch cells under practical operating conditions (high sulfur loading, low E/S ratio, low negative/positive ratio) and developing high energy density Li–S batteries. In 2014, Hagen and colleagues published the first study on Li–S pouch cells, where they examined ideal E/S ratios to realize high energy density pouch cells [27]. Since then, advanced Li–S pouch cells have been studied extensively and significant progress has been made [35, 36]. Cui’s group first proposed the concept of catalysis for Li–S batteries in 2017 [37]. They found that metal sulfides as activation catalysts could catalyze the oxidation of Li_2_S to S. Electrocatalysts have the potential to address the issues of severe LiPS shuttle effect and sluggish sulfur redox kinetics in Li–S batteries. Consequently, electrocatalyst design has attracted a great deal of attention [11–13, 38–49]. After 60 years of continuous development, now, looking ahead to the future, considerable efforts are still required to bring Li–S batteries to the market, such as sulfur loading > 8 mg cm^−2^, electrolyte to sulfur ratio < 3 µL mg^−1^, negative to positive ratio < 4, carbon content < 5 wt%, lifespans > 500 cycles.

**Fig. 2:**
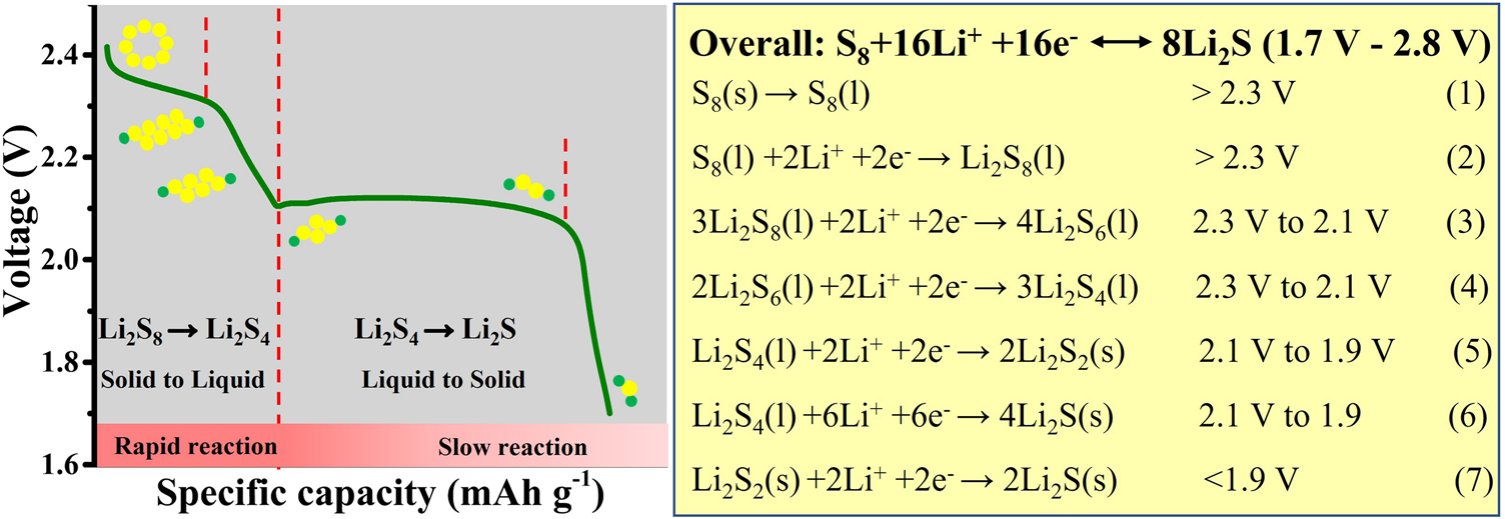
Schematic of the electrochemistry for Li–S batteries

Li–S battery system is regarded as one of the most promising candidates for next-generation  rechargeable batteries because of its low cost (≈ 0.1 $ kg^−1^ for sulfur), high theoretical specific capacity (1675 mAh g^−1^) and high theoretical energy density (≈ 2600 Wh kg^−1^) [52–54]. The mechanism of Li–S batteries is based on chemical transformations rather than intercalation chemistry in Li-ion batteries [55, 56]. For Li–S batteries, the widely accepted reaction mechanism is that during the discharging process, sulfur exists as polysulfides or sulfide of the general formula, Li_2_S_*x*_, where *x* = 8, 6, 4, 2 or 1 (Fig. 2) [57]. In the very first discharge process, solid S_8_ dissolves in the electrolyte to form a liquid state (S_8_(s) → S_8_(l), > 2.3 V). Then, the bonds of liquid S_8_ molecules are broken to combine with Li^+^ to produce long-chain polysulfides as the reaction proceeds (S_8_(l) + 2Li^+^  + 2e^−^ → Li_2_S_8_(l), > 2.3 V). The reaction from S_8_(l) to Li_2_S_8_(l) is spontaneous reaction. Subsequently, in the voltage range of 2.3 to 2.1 V, the highest order polysulfide Li_2_S_8_(l) is reduced to a lower-order polysulfide Li_2_S_6_(l) (3Li_2_S_8_(l) + 2Li^+^  + 2e^−^ → 4Li_2_S_6_(l)); then, the polysulfide Li_2_S_6_(l) is reduced further to soluble products Li_2_S_4_(l) (2Li_2_S_6_(l) + 2Li^+^  + 2e^−^ → 3Li_2_S_4_(l)). The conversion from Li_2_S_8_ to Li_2_S_6_/Li_2_S_4_ is nearly thermodynamical equilibrium. Above is the first discharge voltage plateau corresponding to the conversion of S_8_ to soluble long-chain polysulfides, which provides the theoretical capacity of 419 mAh g^−1^ (25% of the overall specific capacity).

The electrochemical reactions in this phase are shown in Eqs. (1–4):

1 $${\text{S}}_{{8}} \left( {\text{s}} \right) \to {\text{S}}_{{8}} \left( {\text{l}} \right)\quad > {2}.{\text{3 V}}$$

2 $${\text{S}}_{{8}} \left( {\text{l}} \right) + {\text{2Li}}^{ + } + {\text{2e}}^{ - } \to {\text{Li}}_{{2}} {\text{S}}_{{8}} \left( {\text{l}} \right)\quad > {2}.{\text{3 V}}$$

3 $${\text{3Li}}_{{2}} {\text{S}}_{{8}} \left( {\text{l}} \right) + {\text{2Li}}^{ + } + {\text{2e}}^{ - } \to {\text{4Li}}_{{2}} {\text{S}}_{{6}} \left( {\text{l}} \right) \quad {2}.{\text{3 V to 2}}.{\text{1 V}}$$

4 $${\text{2Li}}_{{2}} {\text{S}}_{{6}} \left( {\text{l}} \right) + {\text{ 2Li}}^{ + } + {\text{2e}}^{ - } \to {\text{3Li}}_{{2}} {\text{S}}_{{4}} \left( {\text{l}} \right)\quad {2}.{\text{3 V to 2}}.{\text{1 V}}$$

In the voltage range of 1.9 to 2.1 V, the further reduction of the long-chain soluble polysulfides to short chain insoluble products, Li_2_S_4_(l) + 2Li^+^  + 2e^−^ → 2Li_2_S_2_(s), Li_2_S_4_(l) + 6Li^+^  + 6e^−^ → 4Li_2_S(s). At voltages more negative to 1.9 V, insoluble lithium polysulfide Li_2_S_2_(s) is converted increasingly to insoluble lithium sulfide Li_2_S(s), Li_2_S_2_(s) + 2Li^+^  + 2e^−^ → 2Li_2_S(s). The reduction reaction of Li_2_S_2_(s) to Li_2_S(s) is the rate-limiting step, and the slow kinetics of the reaction is attributed to the low conductivity of Li_2_S_2_/Li_2_S. Above is the second plateau, contributing a theoretical capacity of 1256 mAh g^−1^ at 2.1–1.7 V (75% of the overall specific capacity).

The electrochemical reactions in this phase are shown in Eqs. (5–7):

5 $${\text{Li}}_{{2}} {\text{S}}_{{4}} \left( {\text{l}} \right) + {\text{2Li}}^{ + } + {\text{2e}}^{ - } \to {\text{2Li}}_{{2}} {\text{S}}_{{2}} \left( {\text{s}} \right)\quad {2}.{\text{1 V to 1}}.{\text{9 V}}$$

6 $${\text{Li}}_{{2}} {\text{S}}_{{4}} \left( {\text{l}} \right) + {\text{6Li}}^{ + } + {\text{6e}}^{ - } \to {\text{4Li}}_{{2}} {\text{S}}\left( {\text{s}} \right)\quad {2}.{\text{1 V to 1}}.{\text{9 V}}$$

7 $${\text{Li}}_{{2}} {\text{S}}_{{2}} \left( {\text{s}} \right) + {\text{2Li}}^{ + } + {\text{2e}}^{ - } \to {\text{2Li}}_{{2}} {\text{S}}\left( {\text{s}} \right)\quad < {1}.{\text{9 V}}$$

**Fig. 3:**
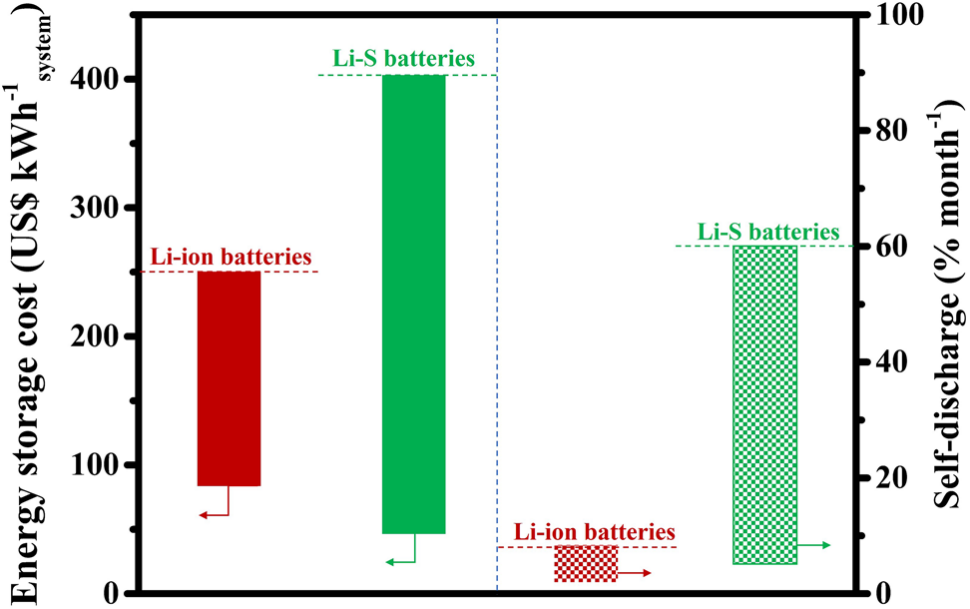
Left: energy storage cost of Li-ion and Li–S batteries [5]. Copyright 2018, Springer Nature. Energy storage cost refers to the cost of the battery pack or system. Right: the upper and lower self-discharge ranges observed in commercial Li-ion and prototype Li–S batteries. Energy storage costs are calculated based on the calculation method listed in Ref. [58]. Copyright 2022, Elsevier

Li-ion batteries are the dominant energy storage technology to power portable electronics and electric vehicles. Therefore, it is essential to compare the energy density, cost, etc., of commercial Li-ion batteries with those of Li–S batteries, which are still under development. The cost of energy storage in batteries varies depending on various factors such as the size of the battery, the manufacturing process and the specific application [58, 59]. Generally speaking, the energy storage cost of Li–S batteries is higher than that of Li-ion batteries. One of the reasons Li–S batteries are more expensive than Li-ion batteries is the use of sulfur as the cathode material. Although sulfur is abundant and cheap, it has low electrical conductivity and is not very stable. To overcome these challenges, Li–S batteries require more complex cell designs and electrolyte compositions, which can increase the manufacturing cost. According to a report by the International Renewable Energy Agency (IRENA) in 2020, the current estimated cost of energy storage for Li–S batteries is around $250–$400 per kilowatt-hour (kWh). In comparison, the cost of energy storage for Li-ion batteries ranges from $80 to $250 per kWh (Left of Fig. 3). However, it is important to note that the cost of Li–S batteries is expected to decline over time as technology continues to improve and production processes become more efficient.

Self-discharge is the loss of battery capacity over time when the battery is not in use. This is due to the internal chemistry of the battery, which slowly discharges even when no load is connected. Li-ion batteries have a relatively low self-discharge rate, typically losing only 1%–5% of their capacity per month when stored at room temperature [5]. This makes Li-ion batteries a good choice for applications that require long-term storage, such as backup power systems and portable electronic devices. In contrast, Li–S batteries have a higher self-discharge rate because of the loss of capacity and self-discharge when sulfur dissolves and LiPS shuttles between cathode, which is mainly concentration-driven. As a result, Li–S batteries can lose up to 20% of their capacity per day when stored at room temperature (right of Fig. 3). This makes Li–S batteries less suitable for applications that require long-term storage, but they can still be used in applications that require frequent charging and discharging cycles, such as electric vehicles and grid-scale energy storage. The self-discharge rate is highly dependent on the depth of discharge and E/S ratio. As the E/S ratio decreases, making LiPS concentration gradient higher, which inevitably results in more severe shuttle and greater self-discharge [60]. Many researchers have proposed employing catalytic host materials to suppress the shuttle effect and thus reduce the self-discharge rate of Li–S batteries [39, 61–63]. However, the investigation of self-discharge behavior at various E/S ratios in Li–S batteries has received less attention compared to the extensive research conducted on the dynamic cycling efficiency and stability of these batteries.

**Fig. 4:**
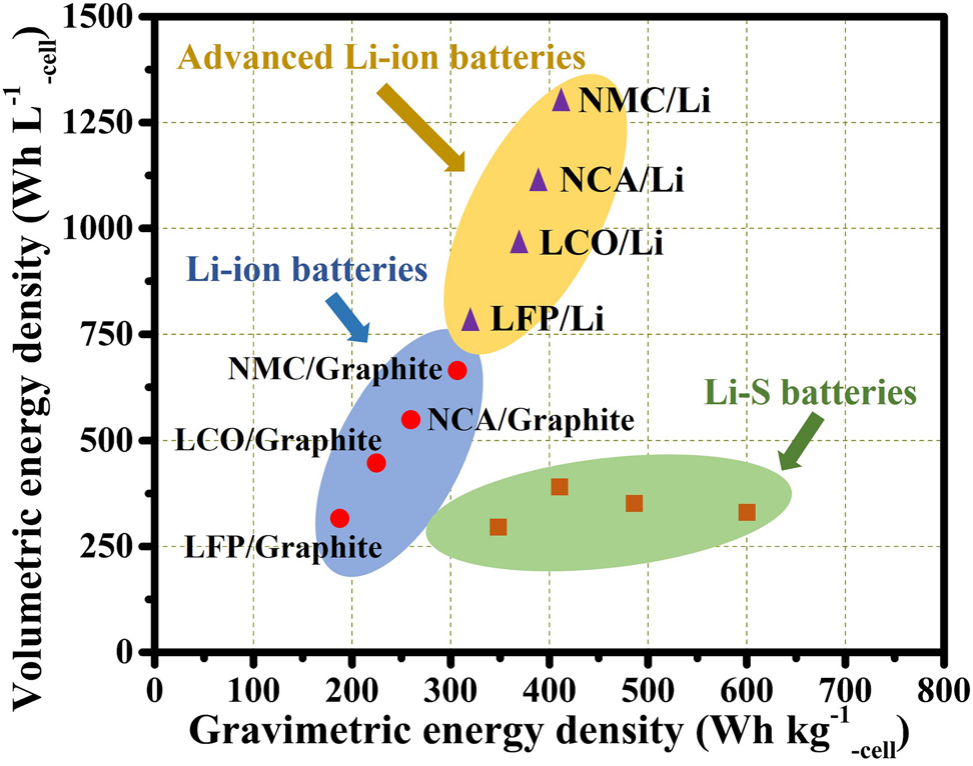
Comparison of gravimetric energy density and volumetric energy density of Li–S batteries, Li-ion batteries and advanced Li-ion batteries. Advanced lithium-ion batteries are those that pair high-capacity lithium transition metal oxide cathodes with silicon and lithium metal, rather than just with graphite anode materials. The data (circles) of LiFePO_4_ (LFP), LiCoO_2_ (LCO), LiNi_0.8_Co_0.15_Al_0.05_O_2_ (NCA) and LiNi_x_Co_y_Mn_1−x−y_O_2_ (NCM) are from commercial Li-ion batteries of CATL, Panasonic, LG and BYD companies, respectively [53]. [4, 68][58] Copyright 2021, Wiley-VCH. The data (triangles) of advanced Li-ion batteries are obtained from Sion Power, with nickel-rich metal oxide as cathode material [67]. For Li–S battery, the data (squares) are collected from Sion Power [67], Oxis Energy, Kaskel’s report [4, 68]. Copyright 2022, Springer Nature. Driving distance was calculated based on the energy density of each battery type, using the calculation method listed in Ref. [58]. Copyright 2022, Elsevier

Comparison of the energy densities of Li-ion and Li–S batteries is represented by the Lagoon diagram (Fig. 4). Current commercial Li-ion batteries provide a driving range of 300 to 600 km with cell-level gravimetric energy densities of 150–265 Wh kg^−1^ for electric vehicles (e.g., Li-ion batteries used in Tesla electric cars have an energy density of about 265 Wh kg^−1^), which is insufficient to realize a drive distance of 500-mile per charge at a reasonable battery pack size to alleviate mileage anxiety [64]. Recent literature has reported that Li-ion batteries with nickel-rich layered oxide cathodes and graphite anodes have energy densities over 300 Wh kg^−1^ [4]. High nickel lithium-ion batteries offer advantages in power output and energy density, but their high cost and limited availability of raw materials may hinder their widespread use in electric vehicles. Due to the multi-electron sulfur redox reaction, Li–S cells can provide a high theoretical energy density of 2600 Wh kg^−1^ and a full cell-level energy density of ≥ 600 Wh kg^−1^. The primary advantage of Li–S batteries over Li-ion is their gravimetric energy density values of 720 Wh kg^−1^ (in the more ideal case) and ∼ 400 Wh kg^−1^ (in most reported) [65, 66]. While cycle stability still struggles at 100 cycles, the gravimetric energy density of Li–S pouch cells has greatly improved to support applications where weight is more crucial than lifespan. For example, the Li–S cell manufacturers such as Sion Power and Oxis Energy announced a new target of 500 Wh kg^−1^ soon after achieving 400 Wh kg^−1^for electric bus, truck and boat applications [64, 67]. Thanks to these fantastic benefits, Li–S battery has the potential to compete with commercial Li-ion batteries in certain applications where high gravimetric energy density is a primary consideration.

Volumetric energy density is another key for practical applications. Higher volumetric energy density means that more energy can be stored in a smaller space, which is particularly important in applications where space is limited [53]. Therefore, it is quite urgent and worthwhile to assess the latest research on Li-ion and Li–S batteries to gain a greater understanding of the volumetric energy density of Li–S batteries [69]. As we can see from Fig. 4, despite their attractive high gravimetric energy density, Li–S batteries are dwarfed by the volumetric energy density of Li-ion batteries. Commercial Li-ion batteries can provide volumetric energy density of 250–750 Wh L^−1^ with graphite anodes, and the values can even surpass 1000 Wh L^−1^ when paired with Li anodes. For Li–S batteries, it is possible to achieve volumetric energy density values up to 1017 Wh L^−1^ under relatively ideal conditions [65]. However, the volumetric energy density of most reported Li–S cells remains within the range of 200–400 Wh L^−1^, which is lower than that of many commercially available Li-ion batteries. The volumetric energy density of Li–S batteries is far below than ideal due to the utilization of cathodes composed of abundant carbon materials. These carbon materials possess high specific surface areas and large pore volumes, leading to a decrease in the tap density of sulfur-based composites. Consequently, the reduced tap density limits the overall volumetric energy density in Li–S batteries. The higher tap density of metal-based compounds compared to lightweight carbon materials makes them advantageous for achieving a higher volumetric energy density in sulfur cathodes [70]. However, despite this potential, the current state of lithium-sulfur batteries still suffers from a poor volumetric energy density. As a result, their volumetric energy density was often excluded in early studies to highlight their superior gravimetric energy density. Therefore, future research works need to trade-offs to balance the gravimetric energy density and volumetric energy density of Li–S batteries. Table 1 summarizes the gravimetric energy density, volumetric energy density, corresponding driving distance and cost for Li-ion, advanced Li-ion and Li–S rechargeable battery systems.

**Table 1:** Comparison of key parameters in different batteries

Battery type	Gravimetric energy density (Wh kg^−1^)	Volumetric energy density (Wh L^−1^)	Driving distance (km)	Cost range ($ kW h^−1^)
Li-ion	190–300	250–860	300–600	70–250
Advanced Li-ion	290–450	750–1300	450–940	≈ 130
Li–S	340–609	250–480	653–1136	36–400

**Fig. 5:**
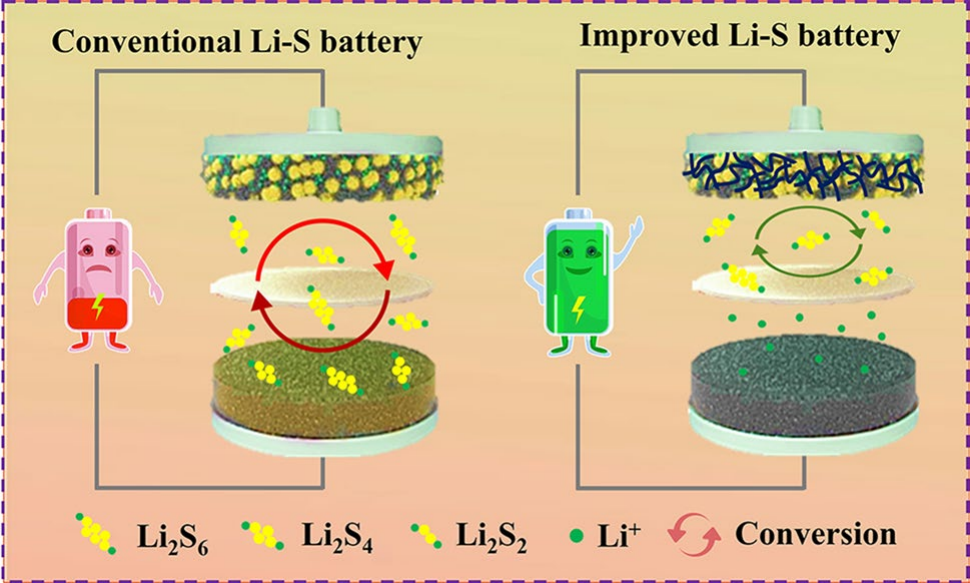
Schematic of the working principles of Li–S batteries and “shuttle effect” of LiPS

The practical utilization of sulfur cathodes in Li–S batteries faces several significant challenges that impact the energy density and cycling stability of the batteries. These challenges include: (1) the electrically insulating character of sulfur and its solid discharge products (Li_2_S_2_/Li_2_S): the low electrical conductivity of sulfur and its discharge products hinders the efficient utilization of sulfur and induces a high redox overpotential; (2) the severe volume change (≈ 79%) during cycling associated with the different densities of S and Li_2_S, leading to mechanical stress and strain on the cathode materials, which can adversely affect their structural integrity and cycling stability; (3) the “shuttle effect” of the soluble lithium polysulfides intermediates, leading to active material loss, decreased Coulombic efficiency and reduced cycling stability of the battery (Fig. 5). In addition to the challenges associated with the sulfur cathode, the use of Li metal anodes introduces safety concerns related to the growth of lithium dendrites. These dendrites can penetrate the separator and induce short circuits, posing safety risks and compromising the long-term performance and cycle life of the Li–S battery.

**Fig. 6:**
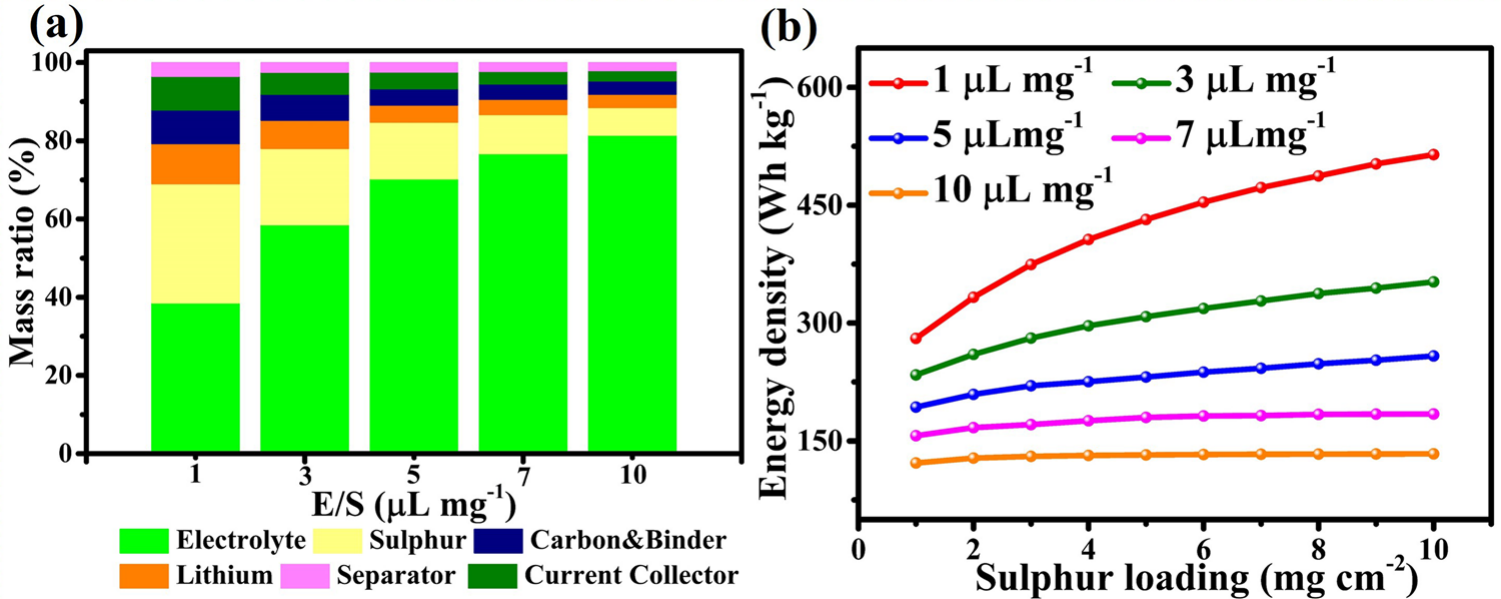
**a** Weight distribution of different components at varied E/S ratios. **b** Energy density of Li–S batteries as a function of varying sulfur loading for different E/S ratios

While various approaches have been used to tackle these issues, demonstrating reversibility with high sulfur loadings (> 15 mg cm^−2^) [71, 72], high sulfur utilization (> 90%) [73–75] and long cycle lifespan (> 2000 cycles) [76, 77], most of these results have been obtained with an excess electrolyte, i.e., E/S ratios exceeding 10 µL mg^−1^. With an E/S ratio of > 10 µL mg^−1^, the energy density of Li–S batteries is compromised, causing them to lose their competitive edge compared to Li-ion batteries [78]. In these conditions, the electrolyte constitutes the largest weight fraction of a Li–S cell (Fig. 6a); electrolyte makes up more than 70 wt% of the overall cell. Such an excess of electrolyte inevitably results in a very low practical gravimetric energy density and a large cost increase, dispatching the two main advantages of Li–S batteries [10, 78–86]. Since high gravimetric energy density can only be achieved at a low E/S ratio, it is essential to minimize the volume of electrolyte for high gravimetric energy density Li–S batteries. As shown in Fig. 6b, the actual energy density of a Li–S cell with an E/S ratio of 10 µL mg^−1^ is below 150 Wh kg^−1^, regardless of the areal sulfur loading. This value is far below what is needed for practical implementations. In conclusion, achieving high energy density in practical Li–S batteries requires a combination of a cathode with a relatively high sulfur loading and a low-volume electrolyte. According to our calculation, a sulfur loading above 5.0 mg cm^−2^ and an E/S ratio below 3.0 µL mg^−1^ are key indicators for Li–S systems to deliver an energy density higher than 300 Wh kg^−1^.

**Fig. 7:**
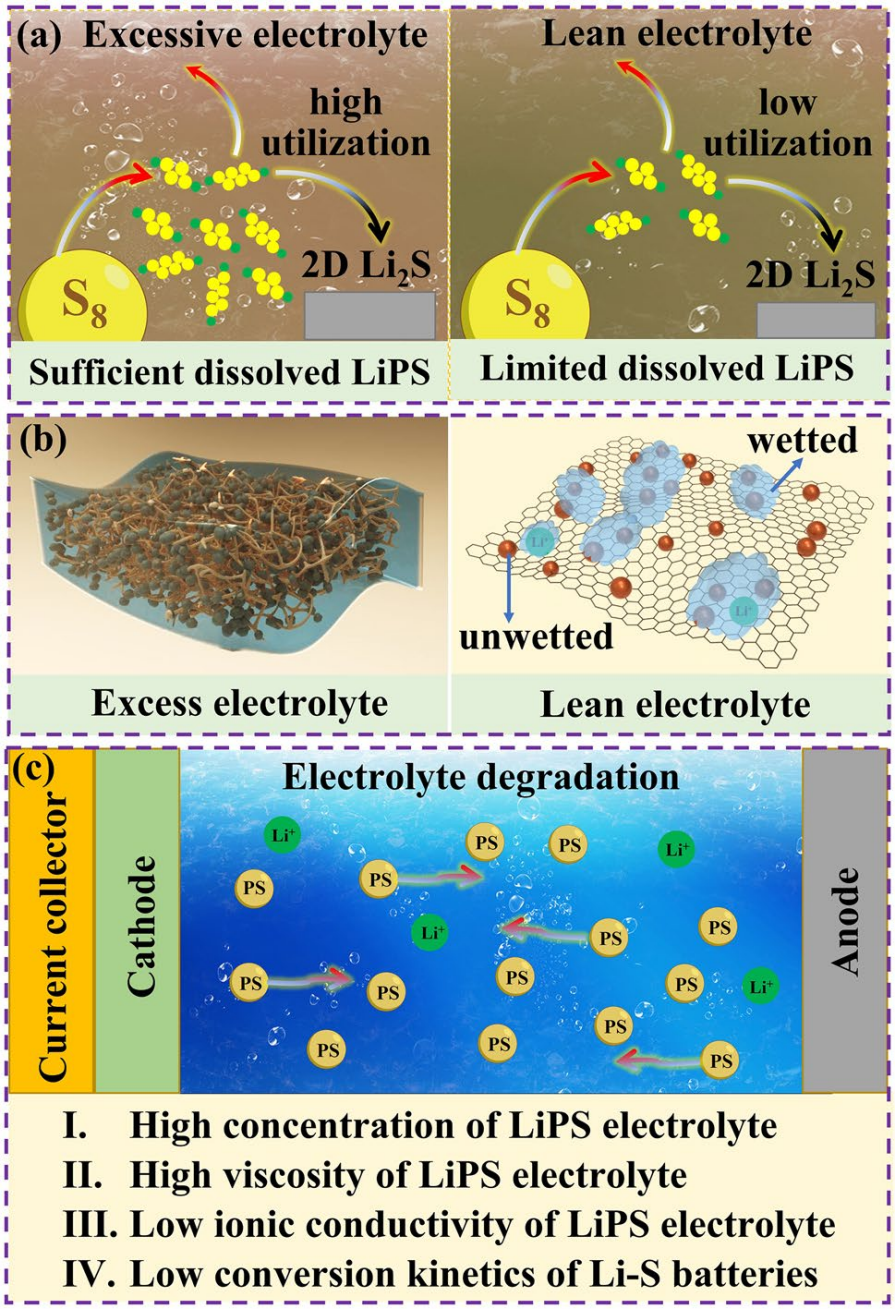
Shortcomings of lean electrolyte: **a** sluggish kinetics of LiPS conversion. **b** Partially wetted surface. **c** Electrolyte degradation and high concentration of LiPS

The use of lean electrolyte conditions causes several major problems in Li–S batteries: (1) sluggish kinetics of the sulfur reduction reactions. As demonstrated in Fig. 7a, excessive electrolyte is necessary to ensure the utilization of sulfur by sufficiently dissolving LiPS. Lean electrolyte conditions result in an inadequate dissolution of LiPS, which reduces kinetics of sulfur reduction reactions and hinders the utilization of sulfur. In addition, due to the insulating nature of sulfur and its discharge products, the sulfur reaction occurs only on the surface of the conductive materials. Therefore, the deposition of undissolved LiPS on the surface of conductive materials can significantly impede the subsequent sulfur reaction. (2) Incomplete wetting of the cathode surface. The electrochemistry of the Li–S cell relies on the redox of soluble LiPS, which in turn depends on rapid ion transport. Ion diffusion is favored when the liquid electrolyte wets the host materials. If the cathode surface is not completely wetted by the electrolyte, the transport of Li ions at the liquid–solid interface is hindered, which severely limits the sulfur redox reaction. As a result, the overall efficiency and performance of the battery are compromised. (Fig. 7b). (3) High LiPS concentration and electrolyte degradation. Lean electrolyte results in an increase in the concentration of LiPS, thereby deteriorating the physical and chemical properties of the electrolyte (Fig. 7c). As the E/S ratio decreases, the ionic conductivity of the electrolyte decreases, and its viscosity increases due to the high LiPS concentration. In addition, the dissolved LiPS interacts with free solvent by solvation and forms clusters with the lithium salt further reducing the ionic conductivity of the electrolyte. The decrease in ionic conductivity leads to increased polarization and poor rate performance.

**Fig. 8:**
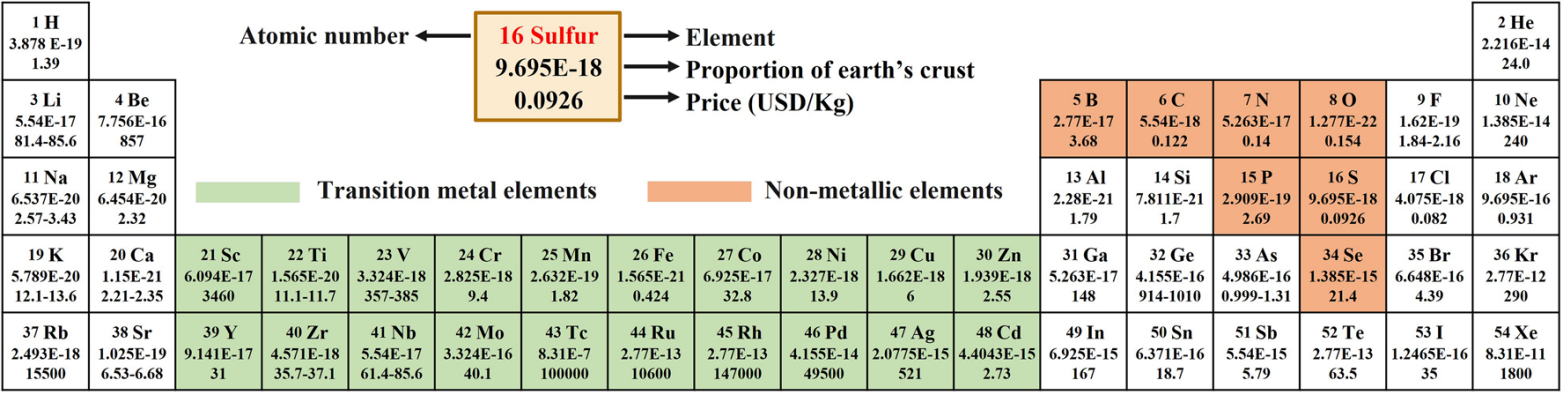
Illustration of the abundance in the Earth’s crust and the price fluctuations of the elements associated with catalysts in Li–S batteries (data from Wikipedia)

In response to the above issues, the rational design of lean electrolyte Li–S batteries focuses on: (1) building composite-based cathodes incorporating a conductive, high surface area and highly porous carbon framework. The carbon framework provides efficient pathways for electron transfer, while the high surface area and porosity promote the diffusion of Li ions and mitigate volume expansion, thus improving the sluggish kinetics of the sulfur redox reactions; (2) bringing in LiPS electrocatalysts. The addition of LiPS electrocatalysts aims to reduce the activation energy required for the sulfur redox reactions to promote the conversion of LiPS and facilitate their electrochemical reactions, leading to high sulfur utilization and accelerated redox reactions. (3) Developing host materials with hydrophilic surfaces. Hydrophilic surfaces have a strong affinity for electrolytes, improving surface wettability and electrolyte penetration into the cathode structure. This enhanced electrolyte penetration enhances the contact between sulfur species and Li ions, promoting the conversion of LiPS and improving the overall reaction efficiency. The extensive use of conductive carbon matrixes effectively reduces the electrode internal resistance, but the physical entrapment based on weak van der Waals forces of carbon-based materials is insufficient to suppress the shuttle effect of LiPS. Therefore, the search for materials that catalyze the conversion reaction of LiPS and have greater adsorption capacity has become a hot research topic in recent years. Polar transition metal-based compounds can form chemical bonds with LiPS, which are based on Lewis acid–base interactions and are stronger than the physical bonds that occur on carbon-based materials. Many transitions metal-based compounds have been shown to accelerate the electrochemical reactions of LiPS [15, 51, 87–92], such as metal nanoparticles (TMs), metal oxides (TMOs), metal chalcogenides (TMCls), metal phosphides (TMPs), metal nitrides (TMNs), metal carbides (TMCs) and metal–organic frameworks (MOFs), single atoms (SAs). Considering the price, abundance, tunable properties, etc., of the elements in the periodic table, transition metal compounds as catalysts for sulfur redox reactions in Li–S batteries exhibit large advantages (Fig. 8), such as (a) Abundance: most of the transition metal compounds used in Li–S batteries are relatively abundant in the earth's crust, making them more accessible and cost-effective compared to other precious metals such as gold or platinum. This makes them an attractive option for industrial scale applications; (b) Tunable properties: the properties of transition metal compounds can be fine-tuned by varying factors such as the metal used, the ligands attached to the metal and the reaction conditions. This allows for greater control over the reaction and improves efficiency. In Li–S batteries, transition metal-based catalytic host materials have shown promising results in improving the sulfur redox reaction and facilitating LiPS conversion by reducing the activation energy. In general, a lower E/S ratio can be employed when using catalytic host materials with high catalytic activity and efficient LiPS conversion. A lower E/S ratio implies a higher concentration of LiPS in the electrolyte, and efficient catalysts can accelerate the conversion of LiPS, enabling a more rapid and efficient utilization of sulfur species.

**Fig. 9:**
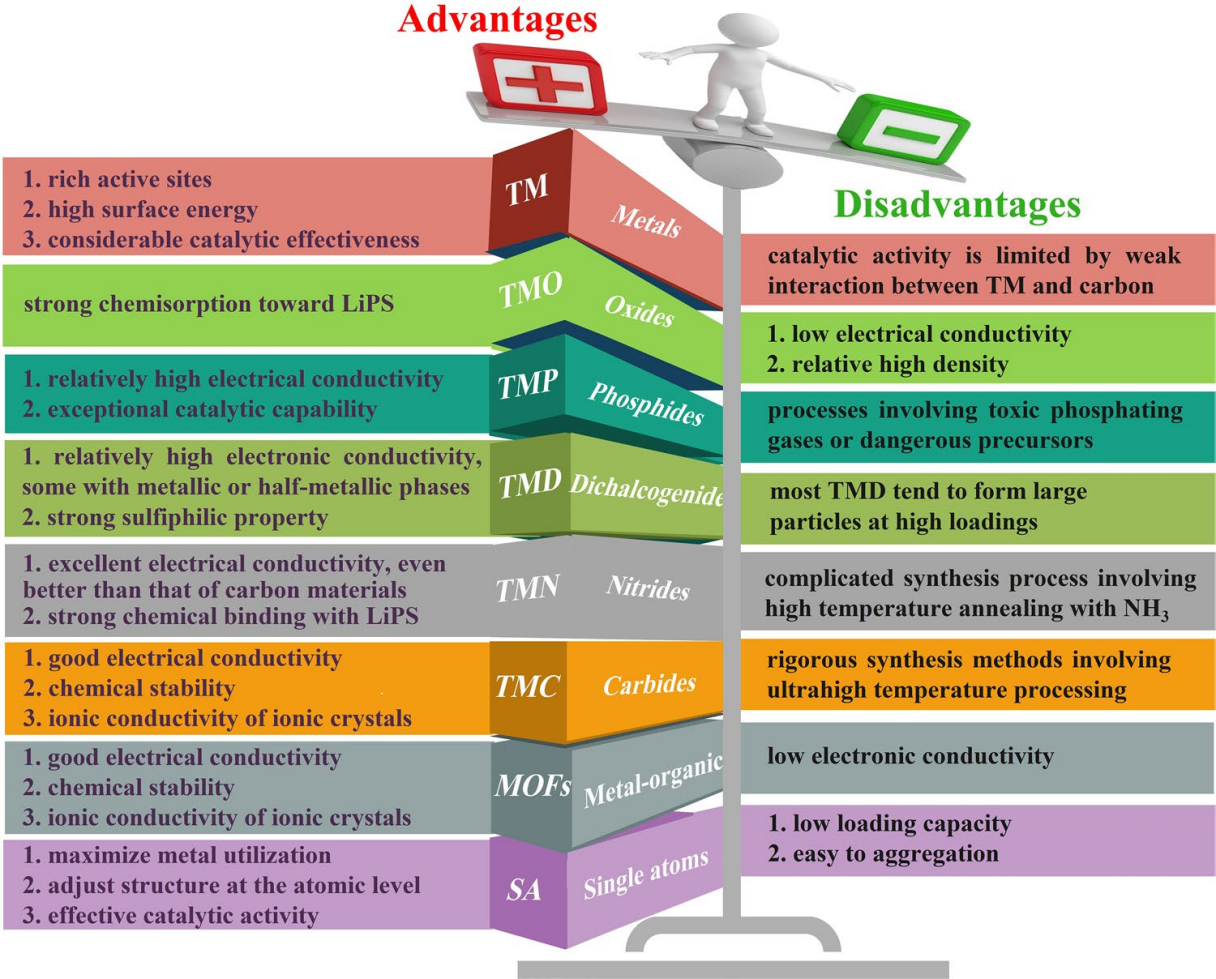
Schematic representation of the advantages and disadvantages of transition metal-based compounds in Li–S batteries

Here, the strengths and limitations of transition metal-based compounds are systematically discussed and presented from a fundamental perspective **(**Fig. 9). This review is focused on recent advances in the use of transition metal-based carbon materials as sulfur hosts for Li–S batteries under lean electrolyte conditions. Firstly, the principles, structure and challenges of Li–S electrochemical conversion under lean electrolyte conditions are discussed in more detail. In addition, the influence of the E/S ratio on the energy density is systematically analyzed. Then, current strategies for cathode hosts and structure design based on transition metal–carbon compounds are addressed. Finally, perspectives on lean electrolyte Li–S cells are presented to guide future research on Li–S batteries.

## Li–S batteries Based on Transition Metal Carbon Materials

Under lean electrolyte conditions, the concentration of dissolved LiPS becomes high, increasing the viscosity of the electrolyte, resulting in incomplete wetting of the cathode, slow ion transport and sluggish redox kinetics of LiPS [93, 94]. At the same time, the high concentration of LiPS increases the LiPS shuttle effect and anode corrosion. To solve these issues, the electrochemical kinetics of LiPS need to be accelerated [78]. Several studies have shown that heterogeneous redox mediators or electrocatalysts can speed up LiPS conversion by decreasing the activation energy [82]. Such a faster adsorption-diffusion-conversion process at the electrode/electrolyte interphase can buffer the severe LiPS shuttle effect while at the same time preventing LiPS aggregation to achieve longer cycle life.

### Metallic Nanoparticles

#### Elemental Metallic Nanoparticles

**Fig. 10:**
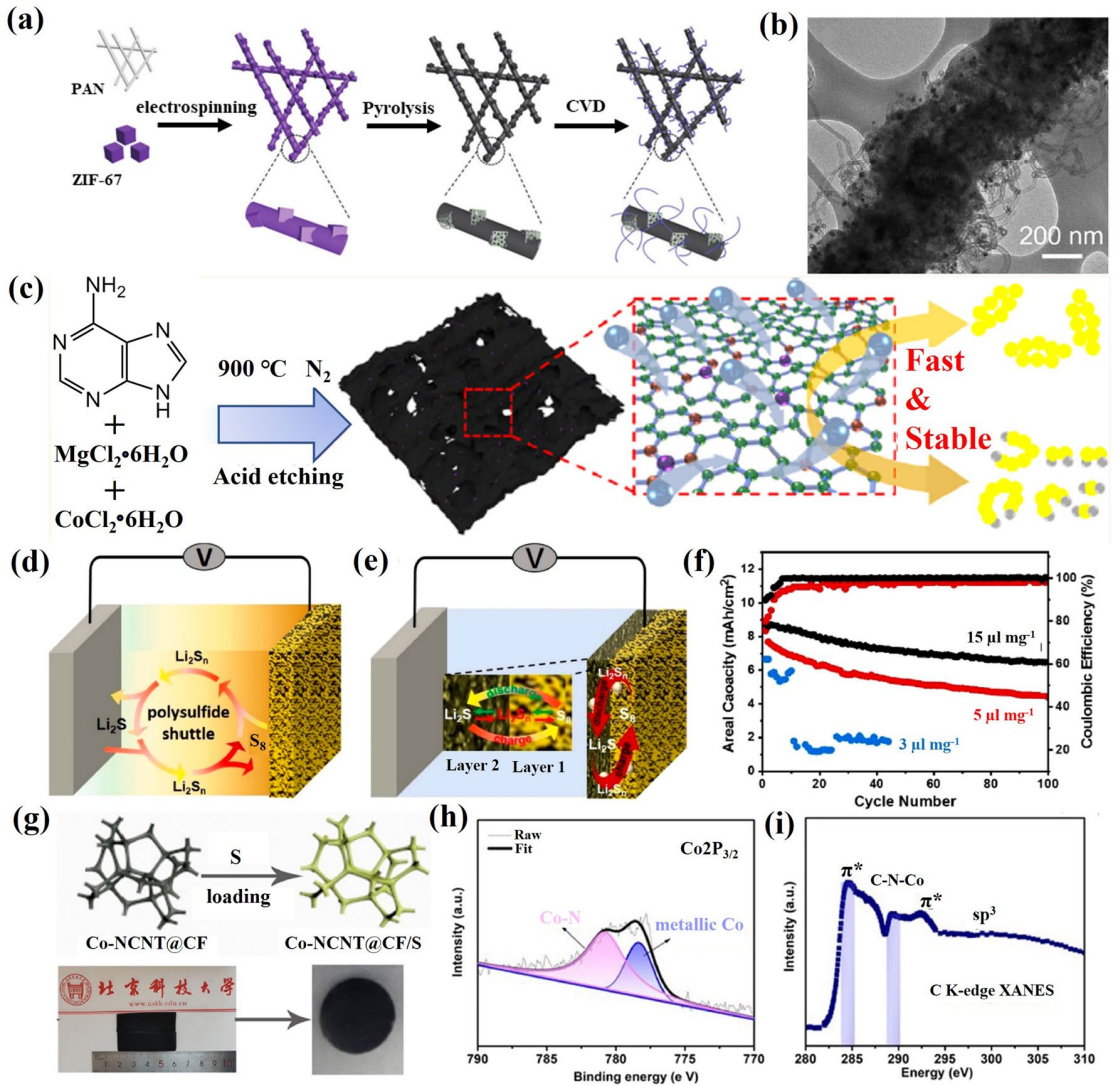
**a** Schematic representation of the fabrication of CPZC fabrics. **b** TEM image of the CPZC fibers [132]. Copyright 2018, Royal Society of Chemistry. **c** Schematic illustration of the synthesis of Co-CNCs [133]. Copyright 2019, American Chemical Society. Shuttle effect of LiPS with **d** a traditional sulfur host and **e** a dual-catalyst layer sulfur host. **f** Cycling performance of the Fe/Co–N@C/S electrodes at 0.4 mA cm^−2^ [93]. Copyright 2019, American Chemical Society. **g** Digital photographs of Co-NCNT@CF/S. **h** XPS of Co 2*p* for Co-NCNT@CF. **i** C K-edge XANES spectra of Co-NCNT@CF [134]. Copyright 2021, Elsevier

Transition metals catalysts are widely applied in chemical reactions, environmental restoration and energy transformation [95–102]. The beneficial effects of transition metal nanoparticles on the performance of Li–S cells depend mainly on the adsorption and electrocatalytic effect of LiPS [29, 103–106]. Beyond noble metal electrocatalysts, some abundant transition metals have been demonstrated to be extremely effective electrocatalysts for LiPS conversion [107–109]. Such nanoparticles are generally anchored to carbon frameworks to maximize particle dispersion and facilitate charge and ionic transport [110–123]. Table 2 summarizes the application of transition metal nanoparticles in lean electrolyte Li–S batteries. Cobalt (Co) nanoparticles embedded into carbon materials as sulfur hosts are the most widely studied for lean electrolyte Li–S batteries [93, 124–131]. Chen’s team designed a stringed “cube on tube” nanohybrid with abundant nitrogen (N) and Co sites as cathode matrices for Li–S batteries under lean electrolyte conditions [132]. The fabrication process of the sulfur host is shown in Fig. 10a. ZIF67 cubes are combined with polyacrylonitrile (PAN) into electrospun nanofibers that are calcined. Then, CNTs are grown using a chemical vapor deposition (CVD) to further construct a hierarchical structure of interconnected and freestanding fibers containing Co particles (denoted as CPZC). The relative contents of N and Co in the as-developed CPZC fabric are estimated to be 7.8 and 13.1 wt%. The batteries based on S@CPZC electrodes delivered an excellent areal capacity of 14.2 mAh cm^−2^ with high sulfur loading (13.5 mg cm^−2^) and lean electrolyte conditions (4.5 mL g^−1^) at 0.05C, which is associated with the high conductivity, and strong physical and chemical adsorption of the hierarchical matrix. Co/N has also been investigated as dual lithiophilic-sulfiphilic sites in Li–S batteries. In this direction, Li’s group designed an interlaced 2D structure including Co/N co-doping (Fig. 10c) [133]. Specifically, metal Co nanoparticles were used as sulfiphilic sites to bind the anions of LiPS (S_*x*_^2−^, *x* = 1–8), and N heteroatoms were utilized as lithiophilic sites to anchor LiPS by interaction with Li^+^. The N and Co atomic ratio was around 6.95 and 0.22%, respectively. This dual adsorption sites allow the host materials to achieve uniform distribution of Li_2_S, associated with a strong trapping ability and a fast conversion kinetics of LiPS. Furthermore, Co electrocatalyst has been shown to have a significant catalytic effect on the LiPS conversion reaction. Ye et al. employed Fe–N and Co–N co-doped carbons as stepwise electrocatalysts to selectively catalyze the conversion of LiPS (Fig. 10d, e) [93]. The relative contents of Fe and Co were estimated to be 4.13 and 3.73 wt%. The authors applied a mixture of sulfur and Fe–N@C as an inner layer and Co–N@C as an outer layer. During the discharging process, long-chain polysulfides formed in the Fe–N@C layer migrated outward and were catalytically reduced to short-chain Li_2_S in the Co–N@C layer. During the charging process, Li_2_S in the Co–N@C layer was catalytically oxidized to long-chain polysulfides and migrated inward into the Fe–N@C layer for further conversion to sulfur. As a result, the batteries based on the dual-catalyst layer as sulfur hosts had high areal capacity with a low E/S ratio of 5. The specific capacity of low E/S (5 µL mg^−1^) is lower than that of high E/S (15 µL mg^−1^) due to lean electrolyte resulting in an insufficient dissolution of LiPS, which hinders the utilization of sulfur and thus leads to a low areal capacity (Fig. 10f). Gu’s group also employed Co nanoparticles and Co-N_x_ co-doped carbon nanotubes embedded in a carbon foam to form a 3D freestanding framework (Co-NCNT@CF) (Fig. 10g) [134]. The high-resolution Co 2*p*_3/2_ spectrum exhibited two peaks of metallic and divalent Co, indicating a strong interaction between Co and N-doped carbon. Additionally, the C K-edge X-ray absorption near-edge structure (XANES) spectrum displayed multiple peaks for a π* transition (284.5 eV) and σ* transition (292.1 eV), with a weak peak (289.7 eV) presented between the π* and σ* transitions, indicating that Co–N–C bonds may have formed in the Co-NCNT@CF composite. These results suggested a high dispersion Co-N_x_ species that provided strong LiPS trapping and promoted the catalytic reaction of LiPS by modifying the electron distribution.

**Table 2:** Representative summary of TMs for the performance of lean electrolyte Li–S batteries

Cathode materials	S loading (mg cm^−2^)	S content (%)	Capacity (mAh g^−1^/cm^−2^)	E/S ratio (µL mg^−1^)	Cycle Life (cycle)	References
Ni-CF/S nanoparticles	5	64	~ 900 mAh g^−1^ at 0.1C	5	150	[103]
Co-NCNT@CF/S nanoparticles	7	–	4.34 mA cm^−2^ at 0.1C	5	After 100	[134]
Co/CNTCNF/PS nanoparticles	5.1	75.2	3.9 mAh cm^−2^ at 0.2C	6	After 300	[132]
Co/CNTCNF/PS nanoparticles	9.2	75.2	6.5 mAh cm^−2^ at 0.2C	6	50	[132]
Mo/CNT/PS nanoparticles	7.64	–	4.75 mAh cm^−2^ at 0.2C	8	After 100	[95]
S/Co-NC@TpBDMe_2_ nanoparticles	5.71	69.5	4.53 mAh cm^−2^ at 0.2C	6.1	After 50	[135]
WIT-Co/S nanoparticles	5.6	80	4.8 mAh cm^−2^ at 0.2C	5	After 100	[102]
Co@NC/S nanoparticles	10.73	–	6.74 mAh cm^−2^ at 0.2C	5.9	120	[112]
S/Co@N-HCMSs	5.1	90.52	5 mAh cm^−2^ at 0.1C	10	50	[136]
Cu-Mo@NPCN/6.5S nanoparticles	10.3	85	906 mAh g^−1^at 0.2C	10	150	[137]
Fe–N/Co–N@C nanoparticles	6.8	–	1316 mAh g^−1^ at 0.5 mA cm^−2^	4	600	[122]
Co-Bi/rGO-S nanoparticles	4.2	72	700.8 mAh g^−1^ at 0.2C	6	10	[111]
Fe–Ni/S alloy	4.1	–	1160 mAh g^−1^ at 0.1C	8	200	[138]
Fe–Ni/S alloy	6.4	86	6.1 mAh cm^−2^ at 0.1C	8	100	[138]
S/HEA-NC alloy	27	–	868.2 mAh g^−1^ at 0.45 mA cm^−2^	3	10	[139]
S/CNC Ni-Pt/G alloy	8.8	75.6	664.9 mAh g^−1^ at 0.03C	5	40	[140]

**Fig. 11:**
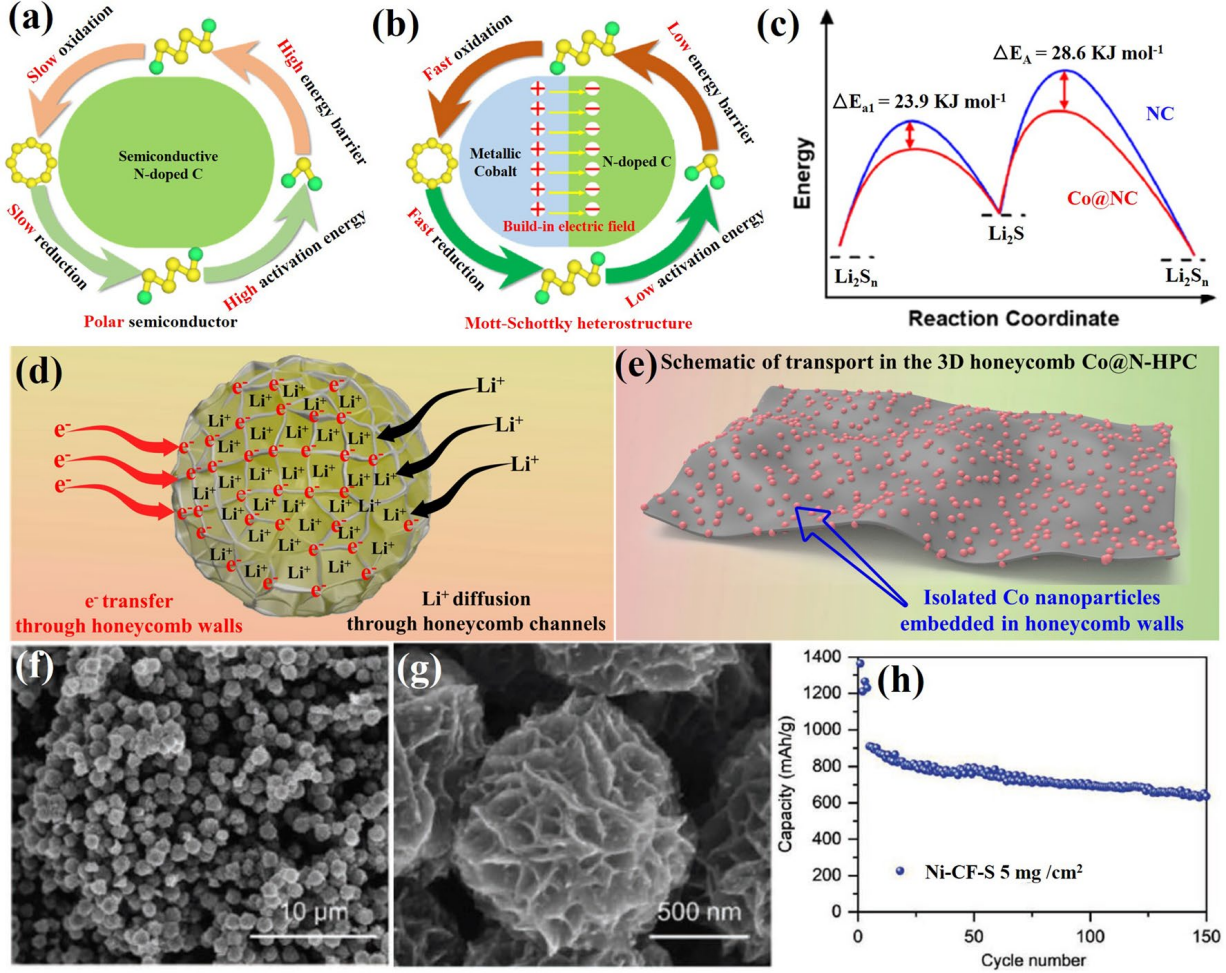
Scheme of **a** an N-doped carbon (NC) semiconductor and **b** a Co@NC Mott-Schottky heterostructure. **c** The activation energies (*E*_a_) for Li_2_S formation and dissolution in NC and Co@CN [112]. Copyright 2021, American Chemical Society. **d** Illustration of electron transfer and ion diffusion in the 3D N-doped honeycomb porous carbon. **e** Schematic illustration of honeycomb walls containing evenly dispersed cobalt nanoparticles [141]. Copyright 2022, Springer Nature. **f**, **g** SEM images of carbon flower with Ni nanoparticles (Ni–CF). **h** The cycling performance of Ni–CF/S electrodes with a sulfur loading of 5 mg cm^−2^ and low E/S ratio of 5 μL mg^−1^. The batteries were cycled at 40C at 0.1C after being tested initially at 0.05C [103]. Copyright 2021, Wiley-VCH

Combining a semiconductor and a metal to form a Mott-Schottky effect may induce interfacial electronic interactions that enhance the catalytic activity [112]. However, the use of Mott-Schottky effect for LiPS catalytic conversion chemistry is rarely reported. Sun’s group employed an N-doped carbon (NC) semiconductor matrix to obtain Co@NC heterostructure as Mott-Schottky catalysts and explored its performance on the LiPS redox reaction (Fig. 11a, b) [112]. As shown in Fig. 11b, in the Mott-Schottky heterojunction, electron transmission can lead to charge separation and produce an internal electric field at the interface, accelerating charge transfer and ion diffusion and lowering the activation energy barrier for catalytic conversion reactions. The authors calculated that the difference in activation energy for the rate-limiting step (the reduction of Li_2_S_n_ into Li_2_S) of the Co@NC/S and NC/S cathode was 23.9 kJ mol^−1^ during the discharge process (Fig. 11c). They also calculated an activation energy difference between the Co@NC/S and NC/S cathodes of 28.6 kJ mol^−1^ in the charging process. The decrease in activation energy values revealed enhanced conversion kinetic of the sulfur species with the Co@NC Mott-Schottky catalysts during both charging and discharging. Recently, our group reported a 3D conductive nitrogen-doped honeycomb porous carbon (Co@N-HPC) with isolated Co nanoparticles as cathode material under lean electrolyte conditions to study the electrochemical performance in Li–S cells (Fig. 11d, e) [141]. The relative contents of N and Co in Co@N-HPC were estimated to be 6 and 6.1 wt%. Just like natural honeycombs, the 3D honeycomb structure contains multiple channels and cobalt nanoparticle-embedded porous walls (carbon nanosheets), which are beneficial for fast Li^+^ ion diffusion and electron transfer. Active sites for bonding with sulfur species are provided by the widely dispersed Co nanoparticles embedded in the N-doped carbon framework and the formation of Co–N–C coordination centers.

Apart from Co nanoparticles, carbon materials decorated with nickel (Ni) nanoparticles have also been explored as electrocatalysts for lean-electrolyte Li–S batteries [142]. Bao et al. designed a carbon flower structure decorated with Ni nanoparticles as a reliable sulfur host to inhibit the LiPS shuttle and promote the reaction kinetics under a low E/S ratio of 5 μL mg^−1^ [103]. As shown in Fig. 11f, g, Ni nanoparticles are encapsulated in porous carbon with a flower shape, resulting in a short ion transfer distance. The authors have verified that 3.76 wt% of Ni was incorporated onto the CF. This flower-shaped structure has a small pore size (below 10 nm) and a high specific surface area (above 3300 m^2^ g^−1^). This particular morphology facilitates the penetration of the electrolyte and shortens the ion diffusion distance. As a result, the cycle performance shown in Fig. 11h indicates that the Ni-CF/S sulfur cathode with a high sulfur loading of 5 mg cm^−2^ and low E/S of 5 μL mg^−1^ maintains 87% of its initial capacity value after 50 cycles at 0.1C.

#### Alloy Nanoparticles

**Fig. 12:**
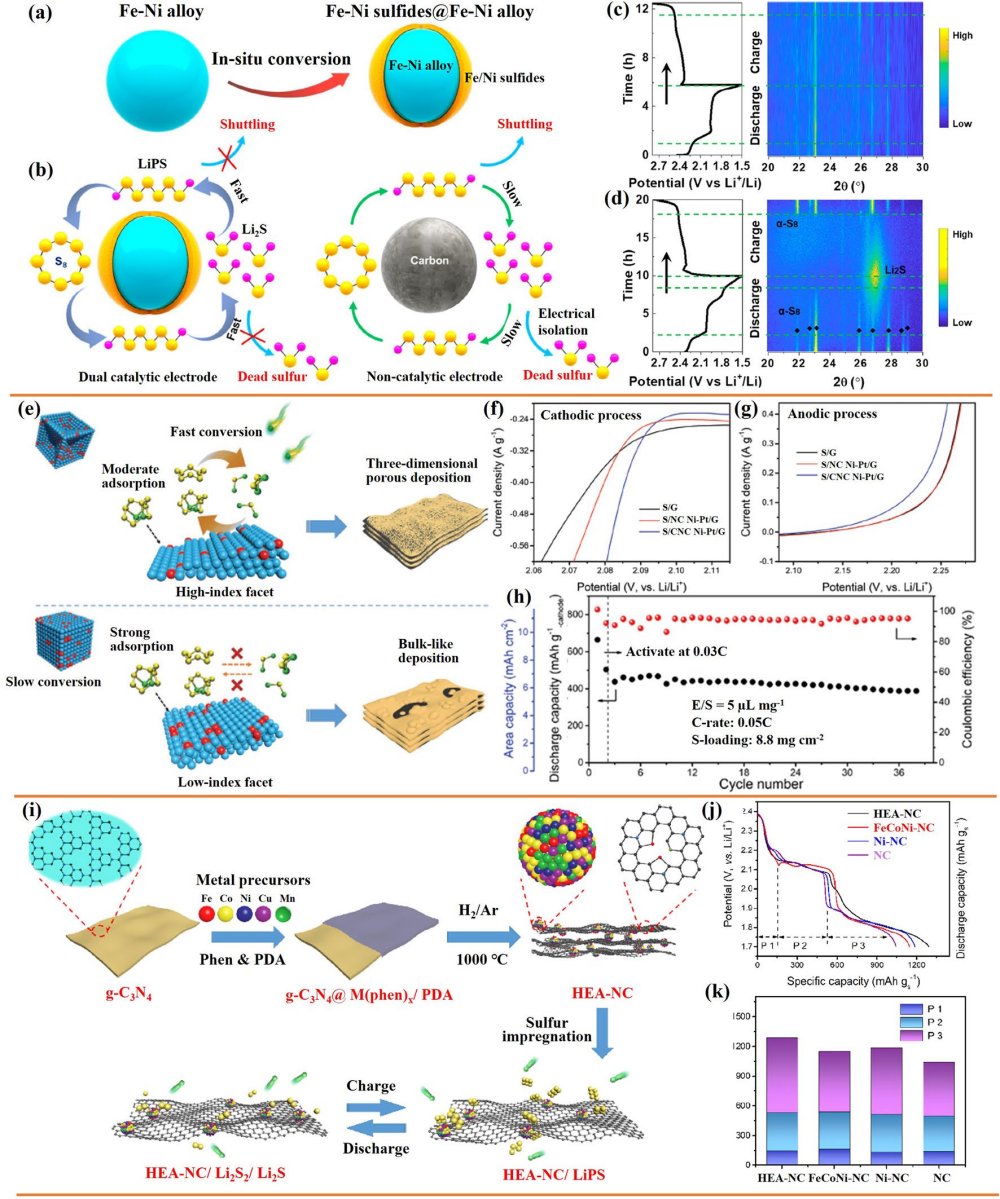
**a** In situ plating of an Fe–Ni alloy with an iron/nickel sulfide layer. **b** Advantages of Fe–Ni alloys. In situ XRD plots of **c** C/S and **d** Fe–Ni/S, the discharge–charge curve is on the left, while the diffraction intensity map is on the right [138]. Copyright 2021, American Chemical Society. **e** Schematic representation of the conversion of LiPS and the precipitation of Li_2_S on Ni–Pt alloy with different facets. **f**, **g** Partial enlargement of the cathodic and anodic processes from the CV curve. **h** Cycling performances of the S/CNC Ni–Pt/G electrodes at 0.05C [140]. Copyright 2022, Wiley-VCH. **i** Schematic representation of the synthesis process of HEA-NC and the promoted conversion reaction of LiPS on the HEA-NC sulfur host. **j**, **k** Li_2_S_6_ test potential curves and the distribution of specific capacity of the various catalysts, P1 (Li_2_S_6_ to Li_2_S_4_), P2 (Li_2_S_4_ to Li_2_S_2_) and P3 (Li_2_S_2_ to Li_2_S) [139]. Copyright 2022, Wiley-VCH

Metal alloys can enhance the catalytic ability of elemental metals through several different mechanisms [143, 144]. Within lean electrolyte Li–S batteries, transition metal alloys show a particular potential associated with their strong catalytic activity for the conversion of sulfur species [145–148]. In this regard, Manthiram’s group engineered Fe–Ni alloys with the hexagonal close-packed (hcp) structure as catalysts through solid-state reactions to enhance the conversion reaction kinetics of LiPS (Fig. 12a, b) [138]. They demonstrated that the efficient catalytic activity of Fe–Ni alloys came from two components: (1) the pristine nanoscale Fe–Ni structure offers active sites and guarantees high catalytic ability; (2) the thin layer plated on the Fe–Ni alloy consists of various sulfurized phases of Fe/Ni sulfides formed in situ during melt-diffusion and with a long duration in the polysulfide-rich environment, which are catalytically active. In situ XRD was used to explore the evolution of the catalysts during the polysulfide conversion process (Fig. 12c, d). The prominent peak in the XRD signal at 27° in the case of Fe–Ni/S cells is caused by the Li_2_S produced in these cells during discharge. In contrast, even near the end of the discharge, the C/S cell does not exhibit Li_2_S peaks. This result suggests that Fe–Ni alloy catalysts accelerate the conversion of LiPS and activate the formation of Li_2_S. The intensity of the Li_2_S peak gradually decreases during the charging process, showing the transformation of Li_2_S into polysulfides bound to the Fe–Ni alloy surface. These results demonstrate the Fe–Ni alloy to be an effective catalyst for the LiPS conversion, providing good cycling performance for Li–S pouch cells with low electrolyte content. Nickel, one of the most used transition metal catalysts, and other Ni-based alloys have also been tested within Li–S batteries. For example, Li et al. explored the use of Ni_2_Co alloys supported on flower-like graphene structures obtained by combining spray drying and high-temperature carbonization, as a bi-service (sulfur host and anode) matrix [149].

The catalytic ability of metal catalysts is highly influenced by the atomic arrangement on their surface and associated configuration. High-index facets (HIFs) have a high density of low-coordinated atoms and are more active than low-index facets (LIFs). Guo’s group designed concave-nanocubic Ni–Pt (CNC Ni–Pt) alloys bound by exposed HIFs as efficient electrocatalysts for Li–S batteries (Fig. 12e) [140]. Compared to the traditional nanocubic Ni–Pt (NC Ni–Pt) alloys with LIFs, HIFs offer moderate adsorption of LiPS and reduce the energy barriers to LiPS conversion, significantly promoting the reaction kinetics of the sulfur species. The S/CNC Ni-Pt/G cathode exhibits the highest cathodic peak onset potential and the lowest anodic peak onset potential (Fig. 12f, g), indicating quicker electrochemical kinetics and significantly reduced polarization by CNC Ni–Pt alloy. As a result, the S/CNC Ni–Pt/G-based Li–S batteries delivered a high initial capacity (665 mAh g^−1^_-cathode_) even with a high sulfur loading of 8.8 mg cm^−2^ and a low E/S ratio of 5 μL mg^−1^ (Fig. 12h). The same group proposed the Fe_0.24_Co_0.26_Ni_0.10_Cu_0.15_Mn_0.25_ high-entropy alloy (Fe–Co–Ni–Cu–Mn HEA) as a catalyst for lean electrolyte Li–S batteries [139]. The highly disordered structure of HEAs at the atomic level with strong lattice distortions often results in high catalytic activity for redox reactions. Fe–Co–Ni–Cu–Mn HEA nanocrystals on nitrogen-doped carbon (NC) substrate (denoted as HEA-NC) were prepared by a reflow process and high-temperature carbonization procedure (Fig. 12i). They demonstrated the Fe–Co–Ni–Cu–Mn HEA nanocrystals have a higher catalytic effect for LiPS conversion than ternary Fe–Co–Ni alloy, while the promoted electrochemical kinetics further leads to a rapid transfer of ions and electrons, thus accelerating the solid–solid (Li_2_S_2_ to Li_2_S) transformation and improving the deposition capacity. Figure 12j, k shows that the HEA-NC electrode contributes the most capacity in the conversion of Li_2_S_2_ to Li_2_S, indicating that the Fe–Co–Ni–Cu–Mn HEA nanocrystals helped to speed up the conversion of Li_2_S_2_ to Li_2_S, achieving high sulfur utilization. Moreover, the cells delivered a discharge capacity of 868 mAh g_-cathode_^−1^ even under both ultrahigh sulfur loading (27.0 mg cm^−2^) and low E/S ratio (3 μL mg^−1^) conditions. This work provides a methodology for researching catalytic host materials to improve the utilization of sulfur in Li–S cells. Table 2 summarizes additional works on alloy nanoparticles for lean electrolyte Li–S batteries.

In general, transition metal nanoparticles and alloy nanoparticles coupled with carbon materials are some of the most promising electrocatalysts to realize high sulfur utilization and long cycle life under lean electrolyte conditions. The dispersion of the metal onto suitable carbon substrates allows for maximizing the surface area and the number of active sites. However, the use of these catalysts is often limited in practical applications due to their high cost, as they may involve expensive noble metals or heavy metals that are not economically feasible.

### Transition Metal Oxides

Transition metal oxides (TMOs) have been used primarily to trap and block the diffusion of highly soluble LiPS at the cathode, thereby alleviating the shuttle effect of LiPS between the cathode and anode [150–161]. A summary of the metal oxide compounds used in lean electrolyte Li–S batteries and their corresponding properties is provided in Table 3.

**Table 3:** Representative summary of TMOs for the performance of lean electrolyte Li–S batteries

Cathode materials	S loading (mg cm^−2^)	S content (%)	Capacity (mAh g^−1^/cm^−2^)	E/S ratio (µL mg^−1^)	Cycle life (cycle)	References
a-Ta_2_O_5_-x/MCN/S	5.6	–	5 mAh cm^−2^ at 0.2C	3.6 mL g^−1^	200	[162]
MnO_2_@rGO/S	4	70	188 Wh L^−1^ at 1.34 mA cm^−2^	4	100	[154]
OV-T_n_QDs@PCN/S	4.8	79.1	736 mAh g^−1^ at 0.5C	4.5	500	[163]
S/Zr_2_N_2_O/NC-6	8.16	77.68	7.2 mAh cm^−2^ at 0.2C	7.79	80	[164]
S@Fe_3_O_4_-NC	3.8	52	~ 1000 mAh g^−1^ at 0.3 mA cm-2	4.2	300	[152]
S-Nb_2_O_5_-*x*/CNTs	6	74	4.8 mAh cm^−2^ at 0.1C	4.4	After 100	[155]
GP/CNT/LNO-V-S	6	–	~ 1200 mAh g^−1^ at 0.05 C	3.8	20	[165]
S/CC@NiCo_2_O_4_	3.5	56	660 mAh g^−1^ at 0.2C	–	After 200	[166]
S/Zr_2_N_2_O/NC-6	8.16	77.68	7.2 mAh cm^−2^ at 0.2C	7.79	80	[164]
LLTO-10/CNT/S	5.2	–	4.5 mAh cm^−2^ at 0.2 mA cm^−2^	5	35	[151]
S/CNT-LDH/Ar	5.5	72	4.4 mAh cm^−2^ at 0.1C	6	After 100	[115]

The significantly different electronegativities of oxygen anions and metal atoms provide metal oxides with strong polarity to chemically interact with polar LiPS or form polythionate complexes that inhibit the shuttle effect of LiPS [167]. However, most TMOs are not suitable for direct service as sulfur hosts owing to their low electrical conductivity and poor catalytic capability. Besides their relatively high weight prevents the use of significant loads of TMOs at the cathode. Thus, TMOs are generally supported on carbon materials to achieve good performance under lean electrolyte conditions [154]. For example, Zhang and co-workers proved that a hyperbranched polymer-coated MOF-derived zirconium nitrogen oxides and N-doping carbon composites (Zr_2_N_2_O/NC-6) possessed rapid Li^+^ transfer, LiPS anchoring and multiple functional sites [164]. Wang’s group constructed sulfiphilic Fe_2_O_3_ nanocrystals restrained in lithiophilic N-doped microporous carbon (Fe_2_O_3_/NMC) to act as sulfur immobilizers for efficient Li–S batteries [168].

**Fig. 13:**
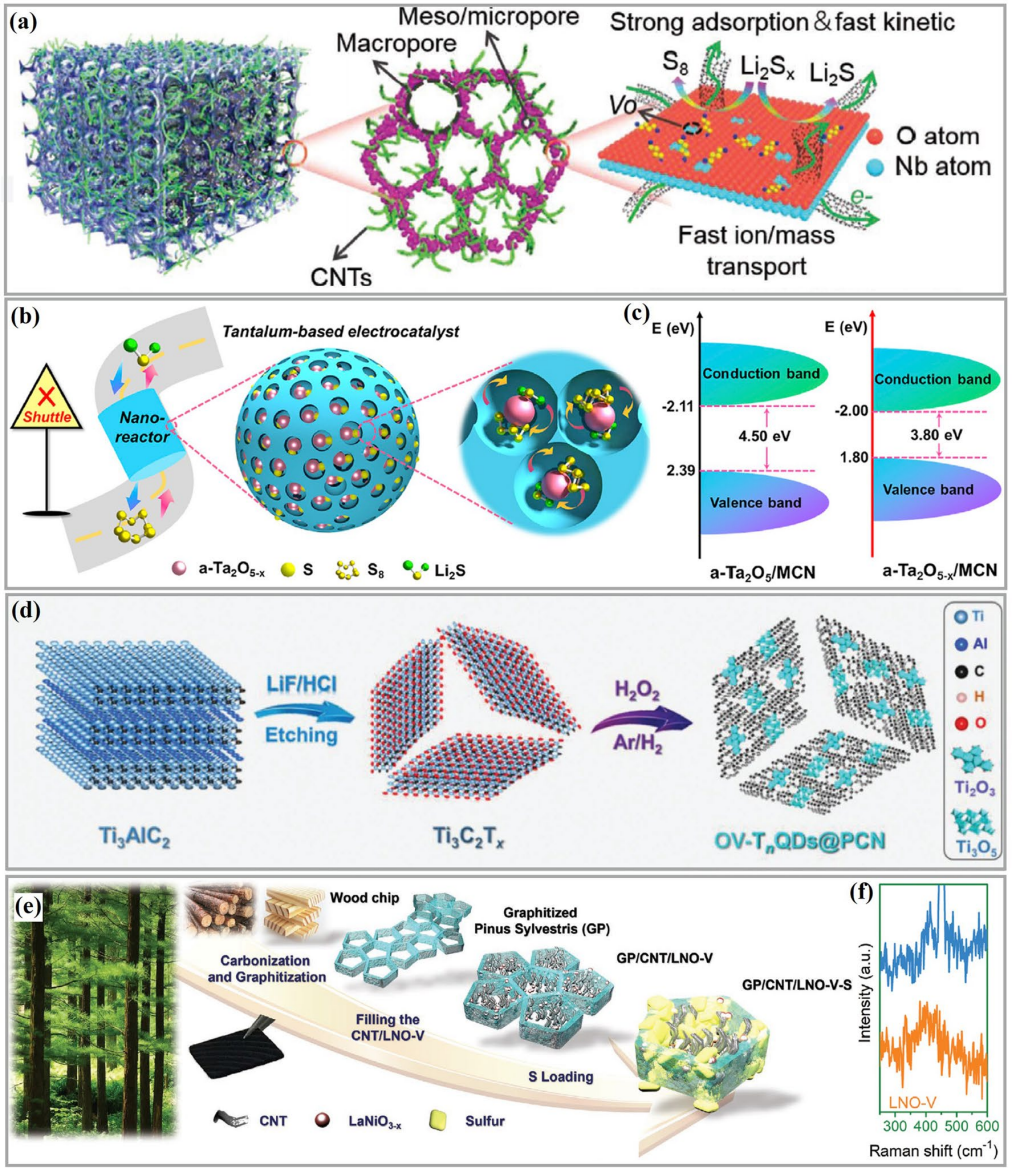
**a** Schematic illustration of Nb_2_O_5−x_/CNTs [155]. Copyright 2020, Wiley-VCH. **b** Illustration of the production of a-Ta_2_O_5−x_/MCN/S. **c** Band graph of a-Ta_2_O5_x_/MCN and a-Ta_2_O_5_/MCN [162]. Copyright 2020, Cell Press. **d** Schematic illusion of the synthesis process of OV-T_n_QDs@PCN cathodes [163]. Copyright 2021, Wiley-VCH. **e** Schematic representation of the preparation of GP/CNT/LNO-V-S materials. **f** Raman spectra of LaNiO_3−x_ [165]. Copyright 2022, Wiley-VCH

Recently, TMOs loaded on carbon materials with oxygen vacancies have been employed as catalytic hosts for Li–S batteries under low electrolyte/sulfur ratios [164, 169, 170]. Chen’s group designed a 3D ordered macroporous framework of niobium oxide (Nb_2_O_5−x_) with oxygen defects as catalytic centers to facilitate the conversion of LiPS [155]. As shown in Fig. 13a, the 3D ordered open and porous framework benefits electrolyte impregnation for rapid Li^+^ diffusion and interfacial exposure to achieve more host–guest interactions. Furthermore, CNTs embedded in the oxygen-deficient Nb_2_O_5−x_ backbone improve the electrical conductivity and catalytic activity. Thanks to these architectural and chemical advantages, S-Nb_2_O_5−x_/CNTs-based Li–S batteries offer good cyclability with increased sulfur loading and lean electrolyte conditions. The same group also proposed Ta_2_O_5−x_ with oxygen vacancies embedded in a “ship in a bottle” nanostructure as a catalyst and adsorber for LiPS reaction and retention (Fig. 13b) [162]. The pores of carbon nanospheres restrain the Ta_2_O_5−x_ nucleation to tune the crystal parameters, thus reducing the length of the Ta–O bond and strengthening the chemical affinity between Ta_2_O_5−x_ and LiPS. In addition, the atomic coordination and electron band structure of Ta_2_O_5−x_ are modified by the oxygen vacancies to increase the electrical conductivity and serve as a catalytic accelerator. Theoretical calculations were used to clarify the effect of oxygen defects on electrical conductivity. As shown in Fig. 13c, the defect-engineered a-Ta_2_O_5−x_/MCN exhibits a smaller band gap than the control sample a-Ta_2_O_5_/MCN, proving the band engineering. This work applies tantalum as a novel catalyst material at practically applicable sulfur loading and electrolyte content, extending the usage of transition metals in Li–S batteries. Quantum dots with ultra-fine particle size (< 10 nm) have their unique advantages compared to nanoparticles (> 10 nm) in Li–S batteries. They offer quantum confinement effects, high surface-to-volume ratios, and most are semiconductive. As a result, these catalysts have demonstrated promising performance in Li–S batteries and have been extensively investigated [171]. For example, Sun’s group demonstrated that oxygen-vacancy-rich Ti_n_O_2n−1_ (Ti_2_O_3_ and Ti_3_O_5_) quantum dots loaded on porous carbon nanosheets can bind LiPS via strong chemisorption and facilitate the transformation of LiPS through a mechanism involving the oxygen vacancies (Fig. 13d) [163]. Owing to the efficient LiPS anchor and high electrocatalytic activity, the cathodes with high sulfur loading (4.8 mg cm^−2^) and low E/S ratio (4.5 μL mg^−1^) exhibited excellent rate performance, with a capacity of 580 mAh g^−1^ achieved even at a current rate of 2C. Jiang’s team utilized NiFe_2_O_4_ quantum dots to build hybrid cathodes with an appropriate tap density (~ 1.32 g cm^−3^) for high-performance Li–S cells. In 2020, the authors applied the unique properties of NiFe_2_O_4_ quantum dots, including excellent chemisorption and catalytic effects on lithium polysulfides, to contribute to the high-rate performance and cycling stability of the NiFe_2_O_4_-based battery [172]. In 2023, the authors further encapsulated NiFe_2_O_4_ quantum dots into nitrogen-rich carbon shells to make microsphere cathodes with high-tap-density (maximum 2.12 g cm^−3^). Such cathodes demonstrated high sulfur utilization and good cyclic behavior [173]. Guo’s team constructed a multiple confined sulfur host by filling graphitized *Pinus sylvestris* with carbon nanotubes and defective LaNiO_3−x_ (LNO-V) nanoparticles (Fig. 13e) [165]. The authors used DFT calculations and experimental results to demonstrate that the unique morphology physically confines LiPS within the microchannel and shows strong chemisorption and high catalytic activity for LiPS due to the spin density around the oxygen vacancies of LaNiO_3−x_. Raman spectra indicated that LaNiO_3−x_ nanoparticles had been successfully loaded into the GP/CNT/LNO-V-S sulfur host electrode (Fig. 13f). The GP/CNT/LNO-V-S-based Li–S batteries showed good discharge capacity at high sulfur loadings and low electrolyte content.

In conclusion, most TMOs possess high surface polarity that facilitates the reaction with polar LiPS. Furthermore, TMOs with oxygen defects provide excellent catalytic activity and stability for the conversion of LiPS. This, together with their simple synthesis makes them a promising sulfur host. However, TMOs need to be combined with carbon materials to increase their electrical conductivity and dispersion.

### Transition Metal Phosphides

Like TMOs, transition metal phosphides (TMPs) are also characterized by notable polarity associated with the differential electronegativity between phosphorous and transition metals. P atoms with high electronegativity can gain electrons from metal atoms and serve as bases for trapping positively charged species [174–176]. In contrast to transition metal oxides, TMPs with the appropriate atomic ratio of metal to phosphide are frequently endowed with a metallic character and even superconductivity [15, 177]. Besides, TMPs can be produced using mild synthesis conditions and have easily tunable electronic configuration and excellent catalytic ability [89, 178, 179]. Thus, not surprisingly, TMPs have been investigated as LIPS adsorbers and catalysts within Li–S batteries [180–182]. A summary of TMPs and their performance in lean electrolyte Li–S batteries is presented in Table 4.

**Table 4:** Representative summary of TMPs for the performance of lean electrolyte Li–S batteries

Cathode materials	S loading (mg cm^−2^)	S content (%)	Capacity (mAh g^−1^/cm^−2^)	E/S ratio (µL mg^−1^)	Cycle life (cycle)	References
S/rGO-CNT-CoP(A)	5.0	67	1006 m h g^−1^ at 0.8 mA cm^−2^	7	100	[183]
CNT-CoP/GO-S	6	72	830 mAh g^−1^ at 0.8 mA cm^−2^	4	50	[184]
CNTs-S@G/CTRu	6.5	80.6	698 mAh g^−1^ at 0.1C	9	After 120	[179]
CF/FeP@C@S	2.5	–	695 mAh g^−1^ at 1C	5	200	[174]
S/SnP0.94@PHCA	6.4	61.75	6.9 mAh cm^−2^ at 0.1 mA cm^−2^	8.6	–	[175]
S/CNT-CoP-Vp	7.7	68	8.03 mAh cm^−2^ at 0.4 mA cm^−2^	5	–	[185]
S/CNT-CoP-Vp	5.5	68	878 mAh g^−1^ at 0.8 mA cm^−2^	7	After 30	[185]
S@Co–Fe–P	1	70	1118 mAh g^−1^ at 0.2C	–	100	[186]
MoP-CNT-10-S	4	72	886 mAh g^−1^ at 0.2C	6	100	[184]
PCPC/NiCoP/S	3.99	72	826.4 mAh g^−1^ at 1C	10	60	[182]
NCNT@Co-CoP@S	10	–	4.4 mAh cm^−2^ at 2C	7	After 100	[187]

**Fig. 14:**
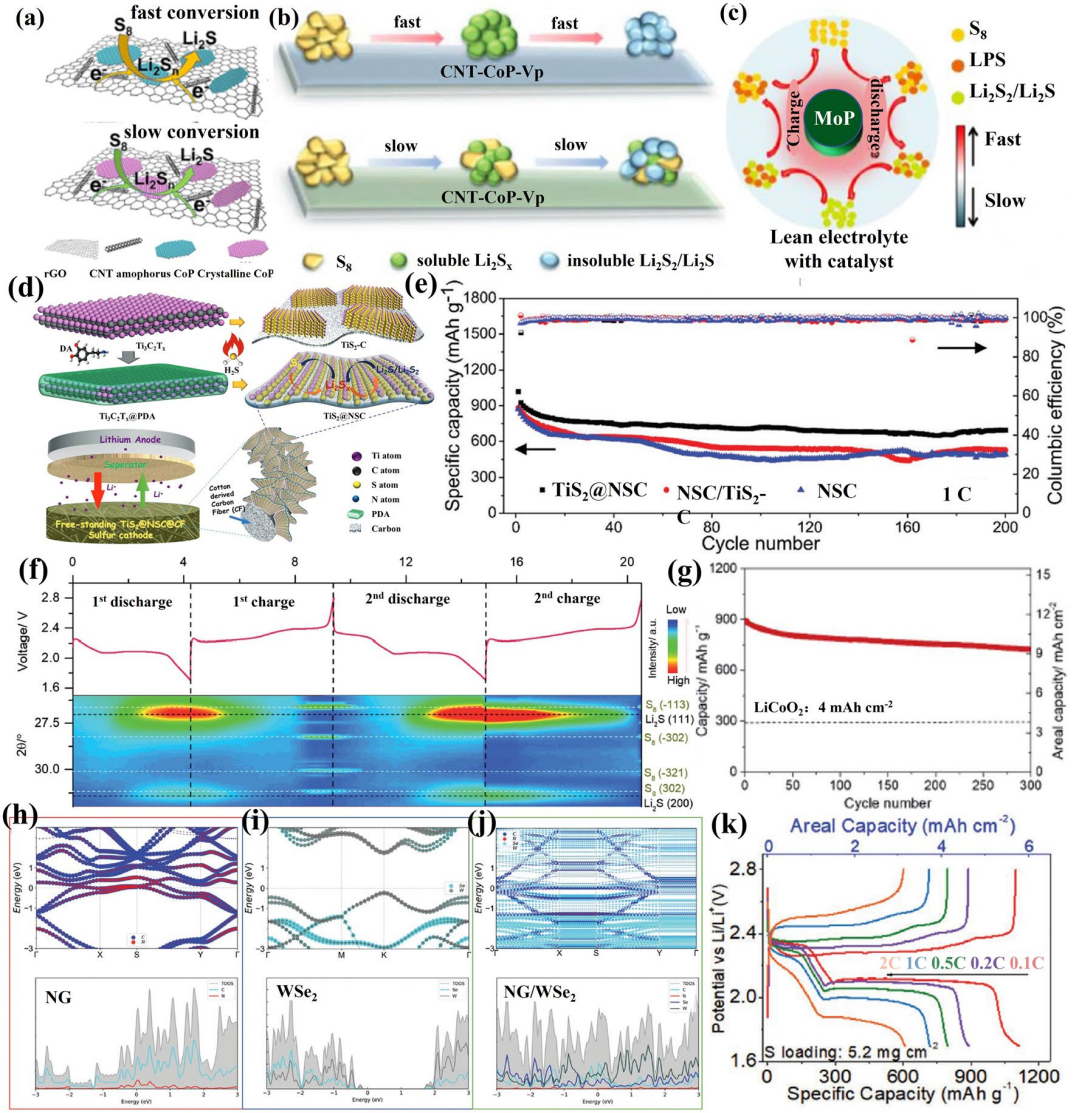
**a** Schematic representation of the reduction processes of LiPS on amorphous CoP and crystalline CoP [183]. Copyright 2021, American Chemical Society. **b** Schematic diagram of the sequential catalytic reaction of LiPS on CNT-CoP and CNT-CoP-Vp [185]. Copyright 2022, Wiley-VCH. **c** Possible reaction routes are described for MoP-catalyzed sulfur cathode under lean electrolyte conditions [184]. Copyright 2018, Wiley-VCH. **d** Illustration of the synthesis of sandwich-shape, monolayer TiS_2_ nanosheets confined within PDA derived N, S co-doped porous carbon. **e** Cycling performance of S/TiS_2_@NSC, S/NSC/TiS_2_-C and S/NSC cathodes at 1C with an E/S of 6 [190]. Copyright 2019, Wiley-VCH. **f** In situ XRD testing of the MoS_2_ ND/porous carbon/Li_2_S_6_ electrode in the first two cycles. **g** Cyclic performance of MoS_2_ ND/porous carbon/Li_2_S_6_ electrodes at a high sulfur loading of 12.9 mg cm^−2^ and a low E/S ratio of 4.6 mL mg^−1^ [191]. Copyright 2020, Royal Society of Chemistry. **h**–**j** HSE06 band structure and DOSs of **h** NG, **i** WSe_2_ and **j** NG/WSe_2_ superlattice. **k** Charge/discharge curves of S@NG/WSe_2_ electrodes at various C-rates with a lower E/S ratio (11.6 mL g^−1^_Sulfur_) [192]. Copyright 2022, Wiley-VCH

Cobalt phosphides, like typical TMPs, exhibit low overpotential for LiPS transitions, suggesting that they could enhance the sluggish redox kinetics of LiPS and improve the rate performance of Li–S cells [188, 189]. Although most studies have concentrated on crystalline cobalt phosphide, the application of amorphous metal phosphides in lithium-sulfur batteries has also been investigated. For example, Sun et al. adopted amorphous cobalt phosphide grown on reduced graphene oxide-multiwalled carbon nanotubes (rGO-CNT-CoP(A)) as the cathode material (Fig. 14a) [183]. Compared to crystalline CoP, amorphous CoP enhances the chemisorption toward LiPS, promotes LiPS conversion and accelerates the nucleation and growth of Li_2_S. In addition, DFT calculations show that amorphous CoP has higher binding energy and a lower diffusion energy barrier to LiPS. Furthermore, amorphous CoP decreases the energy gap and increases the electron concentrations of adsorbed LiPS close to the Fermi level. As a result, combining the advantages of conductive rGO-CNT and the amorphous CoP, the S/rGO-CNT-CoP(A) cathode provides a discharge capacity of 1006 mAh g^−1^ at 0.8 mA cm^−2^ under a sulfur loading of 5 mg cm^−2^ and an E/S ratio of 7 μL mg^−1^.

Comparable to the large interest risen by oxygen vacancies in transition metal oxides and chalcogen vacancies in chalcogenides, recently the influence of phosphorus vacancies on the electrochemical performance of Li–S cells has been a topic of major relevance. As shown in Fig. 14b, CoP with phosphorus vacancies grown on CNT (CNT-CoP-Vp) was used as sulfur hosts with excellent characteristics. The electronic structure of CoP was modified with the introduction of phosphorus vacancies, leading to electron accumulation on Co and P atoms. The defect engineering by phosphorus vacancies enhanced the adsorption and catalytic activity toward polysulfides, resulting in promising electrochemical properties. Thus, a high areal capacity of 8.03 mAh cm^−2^ was realized at 0.4 mA cm^−2^ under lean electrolyte (E/S = 5 μL mg^−1^) and high sulfur loading (7.7 mg cm^−2^) [185]. Besides, this same study revealed the mechanism of phosphorus vacancies for boosting electrochemical performance. The vast majority of cobalt phosphides investigated in Li–S batteries are CoP and Co_2_P. Other valence states of cobalt phosphides still need to be further developed [193]. Molybdenum phosphide nanoparticles supported on CNTs have been also reported as electrocatalysts for stabilizing sulfur cathode under lean electrolyte conditions (Fig. 14c) [184]. Due to the excellent electrocatalytic capability of MoP, the MoP-CNT-10-S electrodes with a low E/S of 4 μL mg^−1^ achieved a capacity of 830 mAh g^−1^ (5.0 mAh cm^−2^) at 0.8 mA cm^−2^.

While it is agreed that the strong chemisorption and excellent electrocatalytic ability of TMPs greatly inhibit the shuttle effect of LiPS, only a few TMPs have exploded so far, and some TMPs with the same composition, but different valence states have not been studied. Furthermore, most of the work on TMPs as electrocatalysts for Li–S cells has focused on the catalytic effect under flooded-electrolyte conditions. The electrocatalytic mechanism in lean electrolyte Li–S batteries should also be further studied.

### Transition Metal Chalcogenides

Transition metal chalcogenides (TMCls) also show a strong chemical affinity with LiPS associated with the electrostatic interaction between the positive metal ion and negative ${\text{S}}_{{\text{x}}}^{2 - }$ within the LIPS. The anchoring ability for LiPS depends strongly on the type of metal ion and to a less extent on the chalcogen, S, Se or Te. Regarding the design of sulfur hosts, TMCls combined with carbon materials have been widely studied as cathode materials in Li–S batteries [191, 194–203]. Table 5 shows an overview of TMCls and their performances in lean electrolyte Li–S batteries.

**Table 5:** Representative summary of TMCls for the performance of lean electrolyte Li–S batteries

Cathode materials	S loading (mg cm^−2^)	S content (%)	Capacity (mAh g^−1^/cm^−2^)	E/S ratio (µL mg^−1^)	Cycle life (cycle)	References
S@NG/WSe_2_	5.2	73.8	885.3 mAh g^−1^ at 0.5C	11.6 mL g^−1^_Sulfur_	350	[192]
S/WSe_1.51_/CNT	9.3	72	5.6 mAh cm^−2^ at 0.1 C	5.5	After 50	[204]
S/WSe_1.51_/CNT	12.7	72	7.7 mAh cm^−2^ at 0.1C	3.9	After 150	[204]
S/TiS_2_@NSC@C	7.7	–	7.9 mAh cm^−2^ at 0.1C	8	100	[190]
S@3DC-CNTs-NiS_2_	6.5	90	5 mAh cm^−2^ at 1.1 mA cm^−2^	5	After 50	[205]
MoS_2_ ND/porous carbon/Li_2_S_6_ cathode	12.9	81	9.3 mAh cm^−2^ at 0.05C	4.6	After 300	[191]
MoS_2_ ND/porous carbon/Li_2_S_6_ cathode	9	–	6.2 mAh cm^−2^ at 0.05C	9.3	After 100	[191]
CC/MoSe_2_@Li_2_S_8_	4	–	1204 mAh g^−1^ at 0.2C	6.2	After 100	[196]
MTQ@3DG/S	4.1	76.2	658.7 mA h g^−1^ at 0.1C	5.5	110	[200]
N-CoSe_2_/S	10.2	70	9.26 mAh g^−1^ at 0.2C	4.4	70	[195]
c-(rGO-CoS_2_)/S	5.6	68	6.67 mAh cm^−2^ at 0.05C	5	40	[206]
S@MoS_2_@CNF	1.0–1.5	72.8	1398 mAh g^−1^ at 0.2C	6	400	[194]
VSe_2_-VG@CC/S	1.4–1.7	70	1025 mAh g^−1^ at 0.5C	4.8	100	[207]
MoSe_2_@C/rGO/S	4.7	73	911 mAh g^−1^ at 0.1C	10	30	[202]
CC@CS@HPP/S	5.6	72	4.1 mAh cm^−2^ at 0.1C	10	After 30	[208]
S@Co_9_S_8_/CNTs-Gr	7.2	75	~ 6 mAh cm^−2^ at 0.5C	10	After 300	[209]
FM@G/87S	–	87	905 mAh g^−1^ at 0.5C	6	120	[210]

Transition metal sulfides are promising sulfur hosts in Li–S batteries due to their strong chemisorption and electrocatalytic activity for LiPS, which facilitates the redox kinetics of LiPS conversion [61, 209, 211–216]. Nanoscale transition metal sulfides frequently show favorable Li–S battery performance, like high specific capacity, long-term lifespan and low redox potential [217]. As the lightest member of transition metal chalcogenides, 2D titanium disulfide (2D TiS_2_) nanosheets have recently received considerable attention. Wang et al. used TiS_2_ nanosheets confined with N, S co-doped carbon (TiS_2_@NSC) as sulfur hosts for high-performance Li–S batteries under lean electrolyte conditions [190]. The sandwich-like ultralight fluffy TiS_2_@NSC was prepared by in situ transformations of Ti_3_C_2_Tx MXene coated with polydopamine (PDA) (Fig. 14d). The introduction of PDA to MXene prevents Ti_3_C_2_Tx restacking and generates unique TiS_2_@NSC structures. This sandwich structure of TiS_2_ nanosheet immobilizes LiPS and provides high electrocatalytic activity for LiPS reduction and lithium sulfide oxidation. As a result, the freestanding S/TiS_2_@NSC cathode shows very high discharge capacity and excellent cycling stability under an E/S ratio of 6 compared to S/NSC/TiS_2_-C and S/NSC cathode (Fig. 14e). CoS_2_ is a semi-metallic crystalline phase possessing a high electrical conductivity of $6.7\;10^{5} \;\Omega^{ - 1} \;{\text{m}}^{ - 1}$ [218]. Li et al. designed dense graphene/CoS_2_/nano-sulfur hybrid paper-like cathodes for high energy density lithium-sulfur batteries under a sulfur loading of 5.6 mg cm^−2^ and an E/S ratio of 5, highlighting their practical applications for power systems [206]. In addition, Zhao’s group reported 0D NiS_2_ nanoparticles on 1D carbon nanotubes (CNTs) supported on a three-dimensional carbon (3DC) framework (3DC-CNTs-NiS_2_) as a sulfur host [205]. Due to the high specific surface area, good conductivity and adsorption and electrocatalysis of NiS_2_ nanoparticles for LiPS, the soft package battery based on the S/3DC-CNTs-NiS_2_ electrodes provided an areal capacity of 5.0 mAh cm^−2^ under an E/S ratio of 5 μL mg^−1^.

MoS_2_, a 2D layered transition metal dichalcogenide, has recently attracted particular attention [219–223]. To date, the MoS_2_ crystal structures covering 1 T, 2H 3R and 1 T' (T-trigonal, H-hexagonal, R-rhombohedral and T'-distorted octahedral) have been widely acknowledged. The use of MoS_2_ as a cathode material in Li–S batteries improves sulfur utilization and cell lifetime [224–227]. Xu and coworkers designed a small amount of 1 T MoS_2_ nanodots (3% of the electrode) as robust electrocatalysts for lean electrolyte Li–S batteries [191]. Computational simulations indicate that the 1 T MoS_2_ surface and Mo-rich edges have a stronger anchoring effect on LiPS, and a lower dissociation barrier of Li_2_S, and faster diffusion of Li ions compared to the 2H phase. Electrochemical characterizations show that 1 T MoS_2_ nanodots promote the trapping of LiPS and accelerate the redox reactions kinetics of LiPS. In situ XRD characterizations shown in Fig. 14f confirm the gradual appearance of Li_2_S during discharge, reaching its maximum intensity at the end of lithiation. The XRD peaks of Li_2_S gradually decrease in intensity during charging, until there are no discernible XRD peaks, after which peaks of monoclinic S_8_ appear. All phase transitions are reversible during the 2nd discharge/charge process. When comparing the in situ XRD results with peer research, Xu and coworkers conclude that MoS_2_ nanodots promote the formation of Li_2_S crystals and the 1 T MoS_2_ nanodots possess a high electrocatalytic capability. As a result, even at a sulfur loading of 12.9 mg cm^−2^ and an E/S ratio of 4.6, the MoS_2_ nanodots/porous carbon/Li_2_S_6_ cathodes had areal capacities of 11.3 and 9.4 mAh cm^−2^ after the 1st and the 300th cycles, which is more than twice that of commercial LiCoO_2_ cathodes (Fig. 14g).

**Fig. 15:**
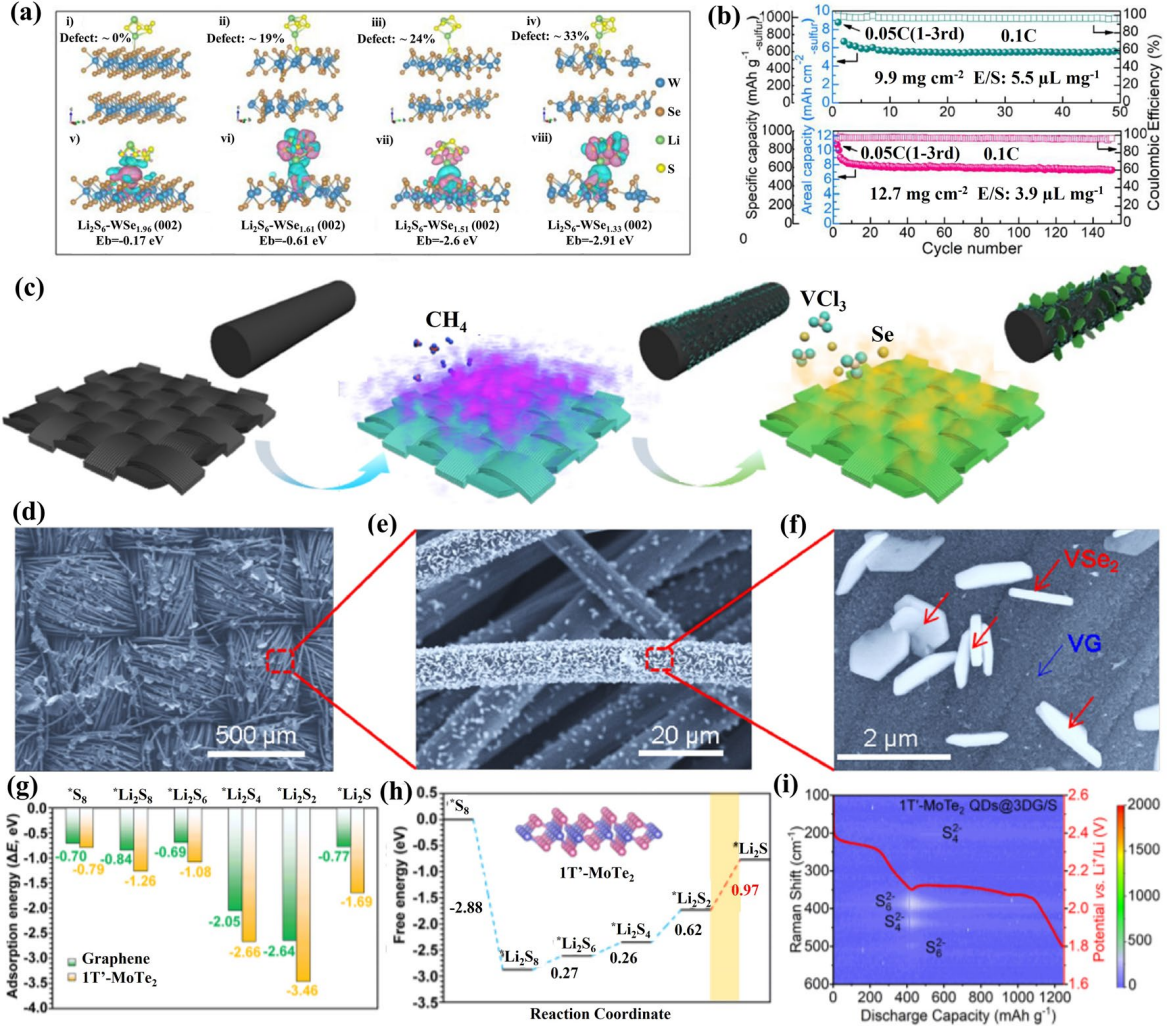
**a** DFT calculations of stable configurations (i–iv) and charge transfers (v–viii), where the pink and cyan represent the spatial regions of increased and decreased charge density, respectively. **b** Cycling performance of the S/WSe_2−x_/CNT cathodes with high sulfur loading and low E/S ratio [204]. Copyright 2022, Elsevier. **c** Schematic diagram of the synthetic process of VSe_2_-VG heterostructure. **d**–**f** Successive magnified SEM images of the VSe_2_-VG heterostructure on carbon cloth [207]. Copyright 2020, American Chemical Society. **g** Adsorption energy comparison between LiPS and 1T′-MoTe_2_ monolayer or graphene. **h** Energy curves for the LiPS reaction on 1T′-MoTe_2_ monolayer. **i** In situ XRD testing of the MoS_2_ ND/porous carbon/Li_2_S_6_ electrode in the first two cycles [200]. Copyright 2021, American Chemical Society

Besides S-based chalcogenides, Se-based chalcogenides are also outstanding sulfur hosts. Cabot’s group applied 2D N-doped graphene/WSe_2_ (NG/WSe_2_) superlattices with an adjustable bandgap to lean electrolyte Li–S batteries [192]. The authors controlled the interlayer spacing from 10.4 to 21 Å by adjusting the annealing temperature. Density functional theory (DFT) is used to identify the electronic band structure and density of states (DOS). As shown in Fig. 14h, NG displays a classic conductor structure and its DOS without a bandgap in the Fermi energy level. Compared to WSe_2_ (Fig. 14i), NG/ WSe_2_ superlattices have no bandgap at the Fermi level (Fig. 14j), indicating high electrical conductivity and rapid ion diffusion in NG/ WSe_2_. As a result, S@NG/WSe_2_ cathodes with a sulfur loading of 5.2 mg cm^−2^ had a discharge capacity of 607 mAh g^−1^ at 2C (Fig. 14k). This work provides a simple strategy for synthesizing superlattice materials and opens up their practical applications in Li–S batteries. Se also is a good catalytic material owing to the presence of Se vacancies. Recently, Guo and co-workers designed a model of 2D WSe_2−x_ with Se vacancies and edge dislocations as a host material to reveal how defects affect catalytic ability [204]. The authors quantitatively regulated the number of defects in WSe_2−x_ by changing the W/Se ratio, and they found that an appropriate number of defects can enable materials with better catalytic performance. The adsorption capacity of the WSe_2−x_/CNT materials with Li_2_S_6_ was investigated by DFT calculations (Fig. 15a): WSe_1.96_ (− 0.17 eV), WSe_1.61_ (− 0.61 eV), WSe_1.51_ (− 2.6 eV), WSe_1.33_ (− 2.91 eV). The adsorbed polysulfide presents improved charge density with sulfur in WSe_1.51_/CNT compared to the co-existence of increasing and decreasing sulfur atoms in WSe_1.61_/CNT and WSe_1.33_/CNT, making it easier for the polysulfide on WSe_1.51_/CNT to trap lithium ions from the electrolyte and complete the cathode reactions. As a result, even at sulfur loadings of 12.7 mg cm^−2^, corresponding to an E/S ratio of 3.9 μL mg^−1^, the areal capacity of S/WSe_1.51_/CNT still remained 7.7 mAh cm^−2^ after 150 cycles at 0.1C (Fig. 15b), indicating a moderate number of Se defects can improve the electrochemical performance of Li–S batteries. Chen’s group designed CoSe with a hierarchical porous polyhedron structure (CS@HPP) as electrocatalyst for promoting the diffusion and conversion of LiPS [208]. The crystal quality and high number of active sites of CC@CS@HP accelerate the catalytic conversion of LiPS and deposition/decomposition of Li_2_S, and batteries based on CC@CS@HPP sulfur as cathode achieve a high areal capacity under a lean electrolyte. Defective VSe_2_ has also been studied as an electrocatalyst for lean electrolyte Li–S batteries. For example, Liu’s group built defective VSe_2_-vertical graphene (VG) nanosheets on carbon cloth (denoted as VSe_2_-VG@CC) as sulfur host for Li–S batteries [207]. As shown in Fig. 15c, it is a two-step conversion process. First, VG is produced on carbon cloth by plasma-enhanced chemical vapor deposition (PECVD) to get VG@CC. Then, by using VCl_3_ and Se as precursors, VSe_2_ nanosheets are grown on the VG@CC support to form VSe_2_-VG heterostructures by van der Waals interactions. The SEM images of the material are shown in Fig. 15d–f. The VSe_2_-VG@CC/S electrodes accelerate the adsorption and conversion of LiPS due to the presence of Se vacancies. Even under harsh operating conditions, such as low E/S ratio (E/S = 4.8 μL mg^−1^) and high sulfur loading (9.6 mg cm^−2^), VSe_2_-VG@CC/S electrodes can achieve an areal capacity of 4.9 mAh cm^−2^ at 0.2C after 40 cycles, which is significantly better than that of commercial Li-ion batteries. Recently, Chen’s group systematically studied the phase transitions (2H, 1T and 1T′) of polar MoX_2_ (X = S, Se and Te) and intrinsic mechanisms for Li–S batteries [200]. Their DFT calculations demonstrate that 1T′-MoTe_2_ has a concentrated density of states (DOS) near the Fermi level, indicating high intrinsic conductivity. The authors also showed that 1T′-MoTe_2_ has high stability. Based on the above analysis, 1T′-MoTe_2_ quantum dots embedded in 3D graphene (MTQ@3DG) were used as electrocatalysts for Li–S batteries. As shown in Fig. 15g, the authors compared the adsorption energies of S_8_ and LiPS on graphene and 1T′-MoTe_2_, which were higher on 1T′-MoTe_2_ than on graphene. In addition, the Gibbs free energy on graphene and 1T′-MoTe_2_ was also evaluated (Fig. 15h). The Gibbs free energy of the rate-limiting step (Li_2_S_2_ to Li_2_S) in graphene is 1.07 eV, while it is 0.97 eV in 1T′-MoTe_2_. In situ Raman spectroscopy demonstrates that the shuttle effect of LiPS is suppressed in the MTQ@3DG/S cells due to the high electrocatalytic ability of 1T′-MoTe_2_ (Fig. 15i). At a relatively low E/S ratio (E/S = 15), the MTQ@3DG/S cathode achieved a discharge capacity of 711.7 mAh g^−1^ after 600 cycles at 1C, with a capacity decay of 0.026% per cycle.

TMCls have been widely studied in lean electrolyte Li–S batteries due to their good electronic characteristics, band position and high number of active sites. Furthermore, metal sulfides have a strong sulfiphilic property for sulfur-containing substances, providing chemical anchoring capability for LiPS. After years of exploration, TMCls are one of the most effective anchors for suppressing the LiPS shuttle effect at low E/S ratios. However, most metal chalcogenides are less conductive than carbonaceous materials and tend to accumulate to form big particles, making it necessary to combine these two materials as cathodes. In addition, the electrocatalytic mechanism in Li–S batteries is not entirely understood. Therefore, more work should focus on the catalytic mechanism of metal sulfides in Li–S batteries.

### Transition Metal Nitrides

**Fig. 16:**
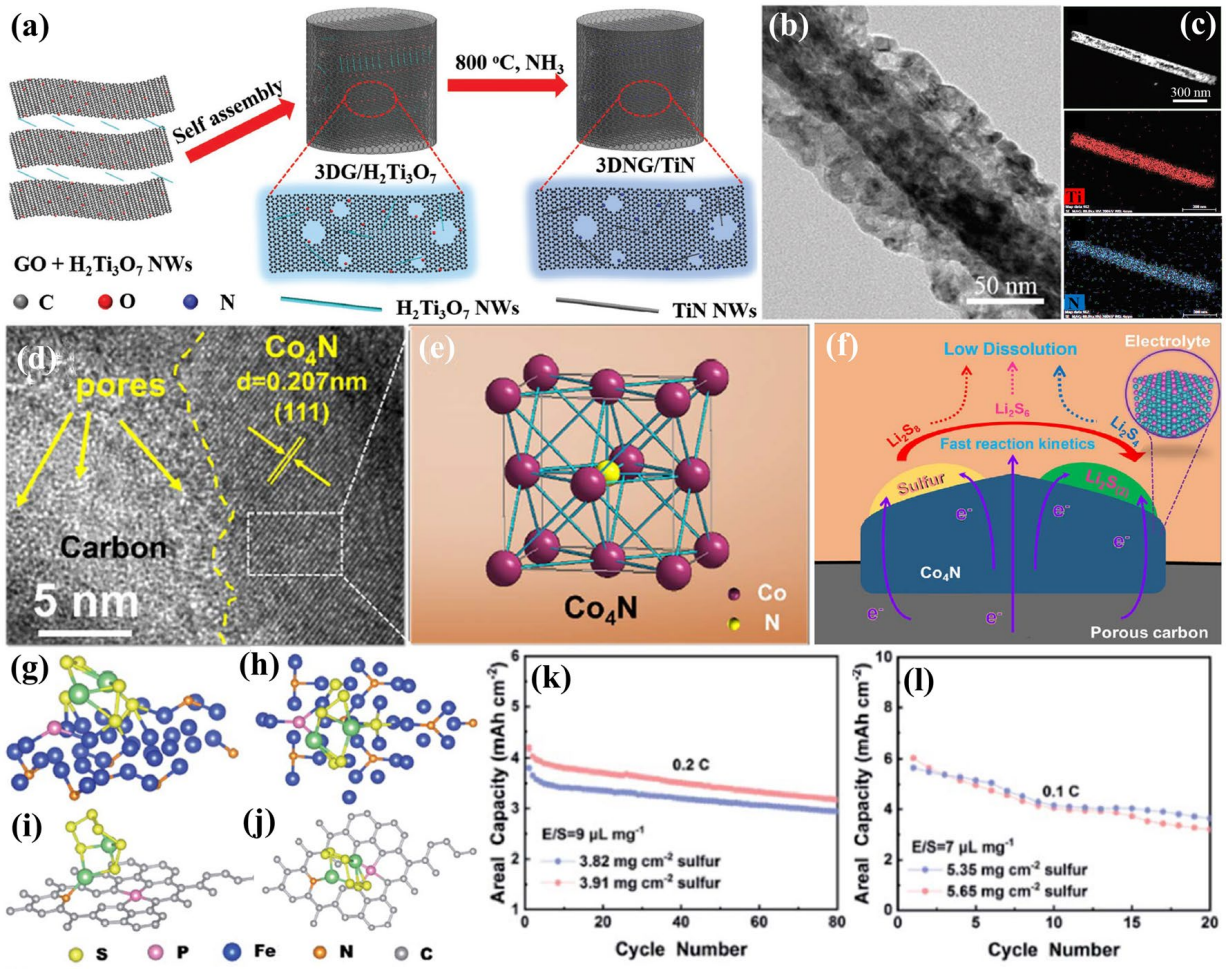
**a** Schematic of the fabrication process of the 3DNG/TiN. **b** TEM image and **c** TEM elemental mapping image of 3DNG/TiN [233]. Copyright 2018, Wiley-VCH. **d** HR-TEM image of CC@Co_4_N-PCNA. **e** Unit cell of Co_4_N structures, **f** chemical absorption and catalytic effect of LiPS on the S/CC@Co_4_N-PCNA cathode schematically [234]. Copyright 2019, Elsevier. **g**, **h** Different angles of the optimized geometry of Li_2_S_6_ on the Fe_4_N (111) surface of P-Fe_4_N. **i**, **j** Optimized geometry of Li_2_S_6_ on the NPG (002) surface at different angles. **k**, **l** Cycling performance of the P-Fe_4_N@NPG/S cathode at 0.2C and 0.1C with lean electrolyte, respectively [235]. Copyright 2021, Royal Society of Chemistry

Transition metal nitrides (TMNs) are another class of material displaying good electric conductivity, polar characteristics and chemical stability [228–231]. Nitrogen atoms are actually an excellent dopant within the carbon matrix because N contains one electron lone pair that can bond to metals to form a coordination center. In addition, the N atoms of TMNs can act as conductive Lewis bases to capture positively charged particles. Furthermore, the electronegativities of nitrogen and sulfur are almost equal enabling the formation of covalent bonds, thus reducing the shuttle effect of LiPS and improving the cycling stability of Li–S batteries [15]. A representative summary of TMN-based compounds for lean electrolyte Li–S performances is listed in Table 6. Titanium nitride (TiN) is the most used TMN in Li–S batteries. Because TiN and other TMNs agglomerate at high loads, leading to low electrochemical activity, they are generally supported on carbon-based materials [232]. For example, Mai’s group designed a 3D nitrogen-doped graphene/TiN nanowires (3DNG/TiN) composite as LiPS anchor for Li–S cells at a relatively low E/S ratio [233]. The TiN nanowires were grown on graphene sheets to form a 3D interconnected network (3DNG/TiN) (Fig. 16a). The TEM image in Fig. 16b shows the rough surface of TiN nanowires with nanoscale pores. TEM elemental mapping showed the homogeneous distribution of Ti and N in a single TiN nanowire (Fig. 16c). The authors also evaluated the binding energy differences of NG, TiO_2_ and TiN with long-chain ${\text{Li}}_{2}{\text{S}}_{n} (n = 4, 6, 8)$. DFT calculations demonstrate greater binding energies between LiPS and TiN, mainly due to the bonding between Li atoms of ${\text{Li}}_{2}{\text{S}}_{n}$ and N atoms of TiN, and S bonding of ${\text{Li}}_{2}{\text{S}}_{n}$ with both N and Ti atoms of TiN. Due to the high conductivity of the 3D porous graphene network and strong chemisorption LiPS on TiN nanowires, the 3DNG/TiN cathode with a high sulfur loading of 9.6 mg cm^−2^ and a relatively low E/S ratio of 10 delivered an ultrahigh areal capacity of 10 mAh cm^−2^ after 60 cycles at 0.5C.

**Table 6:** Representative summary of TMNs for the performance of lean electrolyte Li–S batteries

Cathode materials	S loading (mg cm^−2^)	S content (%)	Capacity (mAh g^−1^/cm^−2^)	E/S ratio (µL mg^−1^)	Cycle life (cycle)	References
FCN111@S/GC	5.36	–	1029 mAh g^−1^ at 1.5 mA cm^−2^	2.5	100	[119]
Co-NbN/rGO/S	5.6	72	3.92 mAh cm^−2^ at 0.1C	8	20	[228]
S/VNQD-HG	4	–	646 mAh g^−1^ at 0.2C	10	After 50	[229]
CC@Co_4_N-PCNA	6.2	35.8	543 mAh g^−1^ at 0.5C	9	After 200	[234]
P-Fe_4_N@NPG	5.65	73	6 mAh cm^−2^ at 0.1C	7	20	[235]
3DNG/TiN	5	–	1230 mAh g^−1^ at 0.1C	10	50	[233]
TiN-TiO_2_/C/S	8	62–66	~ 4.3 mAh cm^−2^ at 0.2C	6.8	After 400	[114]

Cobalt nitrides (Co_4_N) have a robust binding capability to LiPS and catalytically promote the conversion of LiPS. Qiu’s group fabricated Co_4_N nanoparticles embedded in porous carbon nanosheet arrays grown onto carbon cloth (CC@Co_4_N-PCNA) as a self-supported cathode [234]. Co content in CC@Co_4_N-PCNA was about 7.00%. High-resolution TEM images of the CC@Co_4_N-PCNA showed 0.207 nm lattice fringes due to the (111) plane of Co_4_N (Fig. 16d) that displays the atomic structure of closely packed cobalt** (**Fig. 16e). The S/CC@Co_4_N-PCNA electrodes showed stable cycling with a discharge capacity of 543 mAh g^−1^ at 0.5C after 200 cycles at an E/S ratio of 9 and a sulfur loading of 6.2 mg cm^−2^. The excellent electrochemical performance was ascribed to the following (Fig. 16f): (1) The Co_4_N nanoparticles embedded in porous carbon nanosheet arrays on carbon cloth favor the transport of Li^+^ ions and electrons and help keep the structural integrity; (2) the strongly polar Co_4_N suppresses the polysulfide diffusion and reduces the shuttle effect; (3) the catalytic activity of Co_4_N promotes the conversion of polysulfides. Other TMNs have also been studied. For example, Xu and co-workers designed phosphorus-modified Fe_4_N supported by N, P co-doped graphene nanosheets (P-Fe_4_N@NPG) as LiPS anchor [235]. DFT calculations were performed to evaluate the adsorption of LiPS on P-Fe_4_N@NPG for LiPS (Fig. 16g–j). The adsorption energies of Li_2_S_6_ on P-Fe_4_N@NPG and control sample NPG were − 7.02 and − 0.414 eV, respectively, suggesting that P-Fe_4_N@NPG has a much stronger chemical adsorption ability toward LiPS compared to NPG. As a result, the P-Fe_4_N@NPG/S cathode had an initial capacity of 4.19 mAh cm^−2^ under an E/S ratio of 9 μL mg^−1^ and a sulfur loading of 3.91 mg cm^−2^ at 0.2C (Fig. 16k). Even at a lower E/S ratio (7 μL mg^−1^) and higher sulfur loading (5.65 mg cm^−2^), the P-Fe_4_N@NPG/S cathodes delivered an initial areal capacity of 6 mAh cm^−2^ at 0.1C, and it still maintained an areal capacity of 3.2 mAh cm^−2^ after 20 cycles (Fig. 16i). These results indicate the P-Fe_4_N@NPG materials allow high sulfur utilization, inhibit the shuttle effect of LiPS and promote the redox kinetics of LiPS conversion.

Overall, although TMNs have achieved long cycle stability in Li–S batteries, most studies involving TMNs electrocatalysts in Li–S batteries have been assessed with excessive electrolyte. The electrocatalytic ability of TMNs under lean electrolyte conditions needs to be further investigated, which is essential for the design of effective electrocatalysts to improve the electrochemical performance of lean electrolyte cells.

### Transition Metal Carbides and MXenes

Analogous to TMNs, transition metal carbides (TMCs) also possess high conductivity and have active sites that chemically anchor to LiPS [236]. These inherent properties make them suitable trapping materials for LiPS. Typical TMCs like Fe_3_C, Co_3_C and Mo_2_C combined with carbon materials have been used as efficient sulfur hosts for lean electrolyte Li–S cells [237–240]. Within the family of 2D transition metal carbides and nitrides, MXenes have recently gained huge attention in numerous different fields accounting for their superior flexibility, outstanding mechanical strength, metallic conductivity, hydrophilic surfaces and large interlayer channels for ion diffusion. Table 7 summarizes recent investigations on metal carbides and MXenes and their corresponding energy landscape in lean electrolyte Li–S batteries.

**Table 7:** Representative summary of TMCs and MXenes for the performance of lean electrolyte Li–S batteries

Cathode materials	S loading (mg cm^−2^)	S content (%)	Capacity (mAh g^−1^/cm^−2^)	E/S ratio (µL mg^−1^)	Cycle life (cycle)	References
Fe_3_C@FC/S-CF	16.1	60	15.2 mAh cm^−2^ at 0.05C	8	70	[241]
Co_3_C@PNGr-CNT/S	15.6	81	12.33 mAh cm^−2^ at 0.05C	8	40	[242]
Mo_2_C@PCN/S	–	72	~ 450 mAh g^−1^ at 0.1 A g^−1^	10	After 15	[238]
TiC/CNFs@Li_2_S_6_	~ 4.2	66.7–70.4	889 mAh g^−1^at 0.2C	15	110	[243]
VC@NCNTs/S	3.6	71.66	~ 600 mAh g^−1^ at 0.5C	7	100	[244]
Mo_2_C/CHS	5	70	904 mAh g^−1^ at 0.5C	7	200	[236]
S/3De-Ti_3_C_2_-2 MXene	6.1	74.7	~ 5.6 mAh cm^−2^ at 0.1C	5.2	200	[245]
KB/S@Ti_3_C_2_T_x_ MXene	5.6	60	810 mAh g^−1^ at 0.2C	7	100	[246]
IS-MGN@S MXene	4	–	1213 mAh g^−1^ at 0.2C	4.8	50	[247]
T@CP-S MXene	–	70.2	920 mAh g^−1^ at 0.2C	5	60	[248]
S/3De-Ti_3_C_2_-2 MXene	6.1	74.7	~ 3 mAh cm^−2^ at 0.1C	5.2	After 200	[245]

**Fig. 17:**
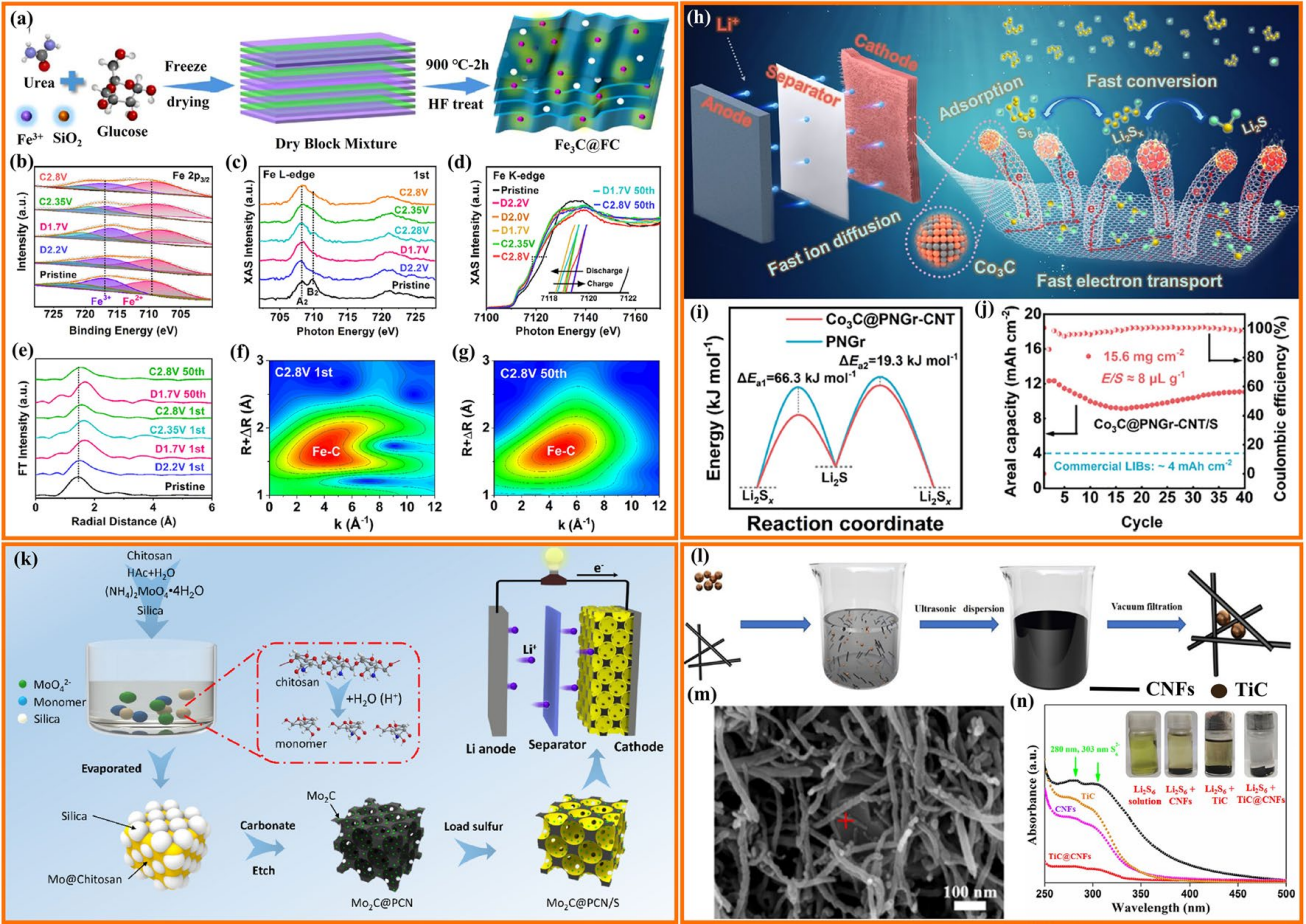
**a** Diagram of the synthesis of Fe_3_C@FC composites. **b** Fe 2*p*_3/2_ XPS spectra. **c** Fe L-edge XANES spectra. **d** Fe K-edge XANES spectrum and **e** corresponding FT-EXAFS spectra of Fe_3_C@FC/S-CF at different states of charge. WT transform contour plots of Fe K-edge after **f** 1 cycle and **g** 50 cycles [241]. Copyright 2022, Elsevier. **h** Schematic diagram of the Co_3_C@PNGr-CNT bidirectional catalytic conversion of LiPS. **i** The activation energy (*E*_a_) of the deposition and decomposition of Li_2_S. **j** Cycling performance of Co_3_C@PNGr-CNT/S cathode with a high-sulfur loading and a relatively low E/S ratio [242]. Copyright 2022, Elsevier. **k** Illustration of the fabrication of the Mo_2_C@PCN/S [249] Copyright 2022, Elsevier. **l** Schematic illustration for the fabrication of TiC/CNFs. **m** SEM image of TiC/CNFs. **n** UV–Vis spectra and digital photographs (inset) of Li_2_S_6_ solution before and after adding CNFs, TiC and TiC/CNFs [243]. Copyright 2022, Springer Nature

Bidirectional TMC-based electrocatalysts have been shown to facilitate the precipitation and decomposition of Li_2_S. For example, Sun’s team designed bidirectional Fe_3_C@foam carbon/S-carbon fiber (Fe_3_C@FC/S-CF) as electrocatalysts to enable the reactive Li_2_S precipitation for Li–S batteries (Fig. 17a) [241]. The 3D porous framework reduces the pathways of Li^+^ diffusion and electron transfer and exposes more active sites of Fe_3_C nanoparticles. The bidirectional Fe_3_C electrocatalysis with good catalytic properties accelerates the deposition and decomposition of Li_2_S. To characterize the chemical interactions between Fe_3_C@FC and sulfur species, XPS and XAFS were performed during the conversion process. Two characteristic peaks of Fe 2*p*_3/2_ located at 709.7 and 716.9 eV correspond to Fe^2+^ and Fe^3+^. The peaks of Fe^2+^ and Fe^3+^ negative shift with increasing discharging depth owing to the electron transfer between Fe and LiPS, and this phenomenon indicates the formation of Fe-S bonds. The associated XPS shifts upward during subsequent charging, showing a reversible Fe valence change (Fig. 17b). The intensity ratio of the multiple structures of the L_3_ edge of the Fe L-edge XANES spectrum is a fingerprint of Fe's different oxidation states (A_2_/B_2_), which decreases/increases gradually during discharge/charge, also proving the reversible reduction/oxidation of Fe in Fe_3_C (Fig. 17c). Moreover, the Fe K-edge XANES spectra, as shown in Fig. 17d, further indicate valence changes of Fe. In agreement with the XPS results, this indicates that Fe_3_C can trap LiPS and achieve a reversible transformation between LiPS and Li_2_S. The corresponding FT-EXAFS spectra show that the Fe–C distance of Fe_3_C widens and narrows from 1.5 to 1.7 Å during cycling due to the breathing behavior caused by the valence change of Fe (Fig. 17e). The reversible behavior was observed even after 50 cycles, which suggests that Fe_3_C retains high catalytic activity during cycling (Fig. 17f, g). As a result, the Fe_3_C@FC/S-CF cells achieved an initial areal capacity of 15.2 mAh cm^−2^ under high sulfur loading (16.1 mg cm^−2^) and relatively low E/S ratio (8 μL mg^−1^) and still maintained 10 mAh cm^−2^ after 70 cycles. Recently, Zhang’s group also proposed a dual-directional catalyst (Co_3_C@PNGr-CNT) as sulfur hosts for bidirectionally catalytic polysulfide conversion (Fig. 17h) [242]. The Co_3_C@PNGr-CNT composites accelerated the deposition and decomposition of Li_2_S, which can be demonstrated by the decreased activation energy of the reduction and oxidation processes (Fig. 17i). As a result, Li–S batteries based on Co_3_C@PNGr-CNT composites enabled a high areal capacity of 11. mAh cm^−2^ after 40 cycles, even with a high sulfur loading of 15.6 mg cm^−2^ and an E/S ratio of 5 (Fig. 17j). These bidirectional catalysts provide new perspectives for the development of high-performance Li–S batteries. Recently, nanosized Mo_2_C electrocatalysts embedded in a porous carbon network (Mo_2_C@PCN) were fabricated by a pyrolysis method (Fig. 17k) [249]. The high conductivity and catalytic property of Mo_2_C@PCN/S, enabled Li–S cells with a specific discharge capacity of ~ 450 mAh g^−1^ at 0.1 A g^−1^ after 15 cycles under an E/S ratio of 5. Shen et al. reported a titanium carbide-modified carbon nanofibers (TiC/CNFs) electrocatalytic membrane for Li–S cells (Fig. 17l) [243]. Figure 17m shows the SEM image of TiC/CNFs, showing TiC nanoparticles on the surface of CNFs with internal cross-linking morphology, which offers fast channels for electron transfer and lithium-ion diffusion. In addition, the UV/Vis measurements reveal that the absorption ability of Li_2_S_6_ by TiC/CNFs is much higher compared to CNFs and TiC, suggesting that TiC/CNFs efficiently bind LiPS (Fig. 17n). The obtained TiC/CNFs electrode exhibits good cycling performances.

**Fig. 18:**
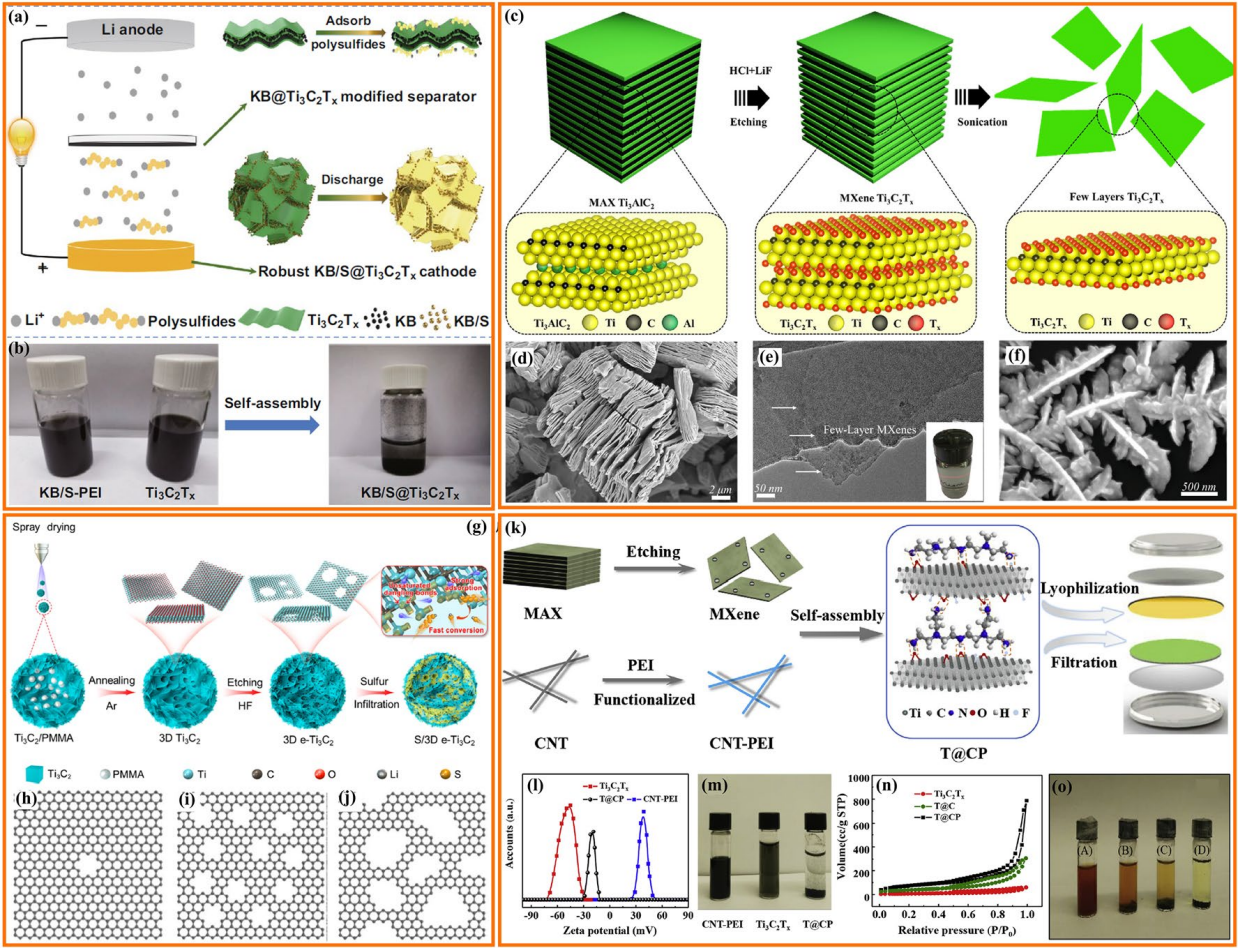
**a** Illustration of the interaction of interwoven KB@Ti_3_C_2_T_x_ with LiPS. **b** Optical images of KB/S-PEI, Ti_3_C_2_T_x_ and KB/S@Ti_3_C_2_T_x_ in an aqueous suspension [246]. Copyright 2020, Springer Nature. **c** Diagram of the synthesis process of the few layers Ti_3_C_2_T_x_. **d** SEM image of Ti_3_C_2_T_x_. **e** TEM image of Ti_3_C_2_T_x_. **f** SEM image of the IS-MGN@S [247]. Copyright 2022, Wiley-VCH. **g** Synthesis schematic of S/3D e-Ti_3_C_2_ microspheres. Morphological evolution diagrams of **h** 3D e-Ti_3_C_2-1_ (120 °C). **i** 3D e-Ti_3_C_2-2_ (150 °C) and **j** 3D e-Ti_3_C_2-3_ (180 °C) [245]. Copyright 2021, American Chemical Society. **k** Schematic diagram of the synthesis of T@CP nanohybrids. **l** Zeta potential of Ti_3_C_2_T_x_ nanosheet, CNT-PEI and T@CP composites. **m** Optical images of CNT-PEI aqueous suspension, Ti_3_C_2_T_x_ and T@CP. **n** N_2_ adsorption–desorption isotherms of Ti_3_C_2_T_x_, T@C and T@CP composites. **o** Optical images of the Li_2_S_x_ solution before (A) and after adding CNT-PEI (B), Ti_3_C_2_T_x_ (C) and T@CP (D) for 6 h [248]. Copyright 2019, Elsevier

MXene, a new class of two-dimensional nanomaterials with hydrophilic surfaces and metallic conductivity, has received significant attention since it was first reported by Gogotsi’s group [248, 250–256]. For example, Zhang and co-workers applied MXene (Ti_3_C_2_T_x_) nanosheets as both cathode, interlayer and modified separator to achieve a long cycle life Li–S batteries with a long cycle life (Fig. 18a) [246]. As shown in Fig. 18b, the interwoven KB@Ti_3_C_2_Tx composite was constructed by self-assembly of the intrinsic negatively charged Ti_3_C_2_T_x_ nanosheets and positively charged Ketjen black/sulfur (KB/S). The structure of KB/S@Ti_3_C_2_T_x_ offers multiple advantages, such as allowing high sulfur content and buffering volume expansion while maintaining good structural integrity and ionic and conductive channels. As a result, the cells achieved a discharge capacity of 810 mAh g^−1^ at 0.2C under sulfur loading of 5.6 mg cm^−2^ and a relatively lean electrolyte of E/S ratio of 7 μL mg^−1^, with a capacity of about 600 mAh g^−1^ after 100 cycles. The aggregation of sulfur causes the low utilization of active materials. Hence, Zhou’s group proposed in situ growth of ultrathin sulfur microcrystals on MXene-based 3D matrices for soft-packaged flexible Li–S batteries [247]. The authors developed an advanced IS-MGN@S electrode by associating the microscale sulfur microcrystal evolution during liquid-phase synthesis and controlling sulfur microcrystal growth on the MXene-graphene-cellulose nanofiber (MGN) matrix (Fig. 18c). Ultrathin sulfur microcrystals consist of a few layers of sulfur atoms, so it is easier to realize improved kinetics than with bulk sulfur. The batteries based on IS-MGN@S cathodes show excellent electrochemical performance, which a capacity of 1213 mAh g^−1^ at 0.2C for a sulfur loading (~ 4 mg cm^−2^) and lean electrolyte content (E/S ratio: 4.8 μL mg^−1^). Chen’s team applied hierarchically porous Ti_3_C_2_ MXene as a sulfur host with desirable properties such as: (1) interlinked backbones provide homogeneous sulfur distribution and avoid restacking of MXene sheets, (2) the high number of active sites and modulation of the d-band center of Ti atoms result in strong LiPS adsorption, (3) unsaturated coordinated Ti on edge sites effectively anchor LiPS, reduce Li^+^ diffusion barriers and accelerate redox kinetics of LiPS [245]. As shown in Fig. 18g, the authors synthesized the hierarchically porous Ti_3_C_2_ MXene with abundant edge sites using PMMA as a template. The authors controlled the number of edges by adjusting the temperature of the spray drying process with a fixed etching time. Figure 18h-j displays the sample morphological changes by changing the spray drying temperature. At a spray drying temperature of 120 °C, a limited number of pores are generated on the HF-etched nanosheets (3D e-Ti_3_C_2_-1). As the temperature is increased to 150 °C, more mesopores are produced, exposing a high number of active edges (3D e-Ti_3_C_2_-2). Multiple large pores are formed when the temperature is increased to 180 °C (3D e-Ti_3_C_2_-3), which disrupts the 3D Ti_3_C_2_ spherical morphology. The micro/mesoporous structure 3D e-Ti_3_C_2_-2 with a high number of edges allows for shortened ion diffusion paths and abundant surface-active sites, which accelerates electrochemical kinetics. Li–S cells based on S/3D e-Ti_3_C_2_-2 cathode deliver a capacity of 5.6 mAh cm^−2^ at 0.1C with a sulfur loading of 6.1 mg cm^−2^ and a low E/S ratio of 5.2 μL mg^−1^. This result demonstrates the importance of edge engineering of 2D layered materials for improving electrochemical performance. In 2019, Guo and co-workers designed an advanced strategy through electrostatic self-assembly of MXene (Ti_3_C_2_T_x_) with polyethyleneimine (PEI)-functionalized CNT for dendrite-inhibited Li–S batteries [248]. Figure 18k illustrates the synthesis process of the Ti_3_C_2_T_x_@CNTPEI (T@CP) composite. As displayed in Fig. 18l, the Ti_3_C_2_T_x_ nanosheets are highly negatively charged with a zeta potential of − 48 mV due to the presence of several functional groups (e.g., –O, –OH and –F). As shown in Fig. 18m, Ti_3_C_2_T_x_ forms stable aqueous solutions. A clear supernatant was observed after adding Ti_3_C_2_T_x_ solution to the CNT-PEI solution, suggesting that CNT-PEI spontaneously adhered to the nanosheet surface and successfully self-assembled. The specific surface area of the T@CP composite was as high as 268 m^2^ g^−1^ compared with Ti_3_C_2_T_x_ (26 m^2^ g^−1^) and T@C (165 m^2^ g^−1^) composite (Fig. 18n). The T@CP composite shows a highly porous structure with pore sizes in the range of 10–80 nm. In contrast, the stacked Ti_3_C_2_T_x_ lacks pores in this size range. These results suggest that MXene, CNT and PEI all contribute to the assembly process. To investigate the ability of these materials to absorb LiPS, CNT-PEI (B), Ti_3_C_2_T_x_ (C) and T@CP (D) were added into equal amounts of Li_2_S_x_ solution (A). As shown in Fig. 18o, the color of Li_2_S_x_ solution after the addition of T@CP was much lighter than CNT-PEI or bare-MXene, indicating T@CP has a strong chemisorption capacity for LiPS. The Li–S batteries based on T@CP as cathode were assembled under lean electrolyte conditions to explore its practical application. The cell achieves a capacity of 920 mAh g^−1^ at an E/S ratio of 5 mL g^−1^.

In general, TMCs have the potential for lean electrolyte Li–S batteries due to their polarity and high electric conductivity. However, the use of TMCs as host materials is still in the early research stage due to the harsh conditions required for their synthesis. Therefore, there is still a need to develop simple synthetic approaches to prepare TMCs with tailored morphologies.

### Metal–Organic Frameworks

Metal–organic frameworks (MOFs) are a relatively new class of crystalline porous materials consisting of metal ions or metal clusters coordinated by organic linkers [257–259]. MOFs are of interest for energy storage, gas separation and catalysis due to their functionalized and tailorable structures [260–269]. Recent studies have indicated that the MOFs can be used as promising cathode materials in lean electrolyte Li–S cells due to their highly porous framework, high number of functional groups and Lewis acid sites [63, 270]. A summary of MOFs compounds and their properties in the Li–S performance is depicted in Table 8.

**Table 8:** Summary of MOFs, SAs catalysts and other transition metal compounds for the performance of lean electrolyte Li–S batteries

Cathode materials	S loading (mg cm^−2^)	S content (%)	Capacity (mAh g^−1^/cm^−2^)	E/S ratio (µL mg^−1^)	Cycle life (cycle)	References
D-UiO-66-NH_2_-4/G MOFs	12.2	89	13 mAh g^−1^ at 0.1C	5	65	[257]
DMAZF/CNTs/sulfur MOFs	5	70	1007 mAh g^−1^ at 0.1C	6	120	[271]
Ni-HHTP@CP MOFs	5.38	–	4.46 mAh cm^−2^ at 0.05C	6.5	25	[272]
CNT@UiO-66-V-S MOFs	5.6	71.4	4.25 mAh cm^−2^ at 0.2C	10	100	[258]
Co–N–C SAC/S single atoms	6	–	825 mAh g^−1^ at 0.1C	4	After 100	[273]
S@Fe–N/MHCS single atoms	5.4	80	6.42 mAh cm^−2^ at 0.1C	8	100	[113]
FeNC/wG single atoms	5.39	–	4.18 mAh cm^−2^ at 0.5C	7.8	50	[101]
S@Co/SA-Zn@N C/CNTs single atoms	5.1	70.7	4.5 mAh cm^−2^ at 0.2C	8	After 100	[274]
SA-Cu@NCNF single atoms	10	66.5	608.8 mAh g^−1^ at 0.2C	6	After 50	[275]
Fe-N_3_C_2_-C single atoms	6.6	–	6.7 mAh cm^−2^ at 0.1C	8	50	[276]
S@Mn/C-(N, O) single atoms	1.1	76	1330 mAh g^−1^ at 0.2C	–	400	[277]
NbB_2_/NPG/S	16.5	75	1030 mAh g^−1^ at 0.1C	6	40	[278]
ZrB_2_/NG/S	8.03	72	8.08 mAh cm^−2^ at 0.1C	8	40	[279]
ZrB_2_/NG/S	4.8	72	1044 mAh g^−1^ at 0.1C	8	132	[279]
G-MgB_2_	9.3	60	≈ 850 mAh g^−1^ at 0.1C	6.5	100	[280]
FeF_2_@rGO/S	12.7	–	12.3 mAh cm^−2^ at 0.1C	6	50	[281]
Nickel-foam@carbon-shell	40	–	41 mAh cm^−2^ at 0.2C	7	100	[282]

**Fig. 19:**
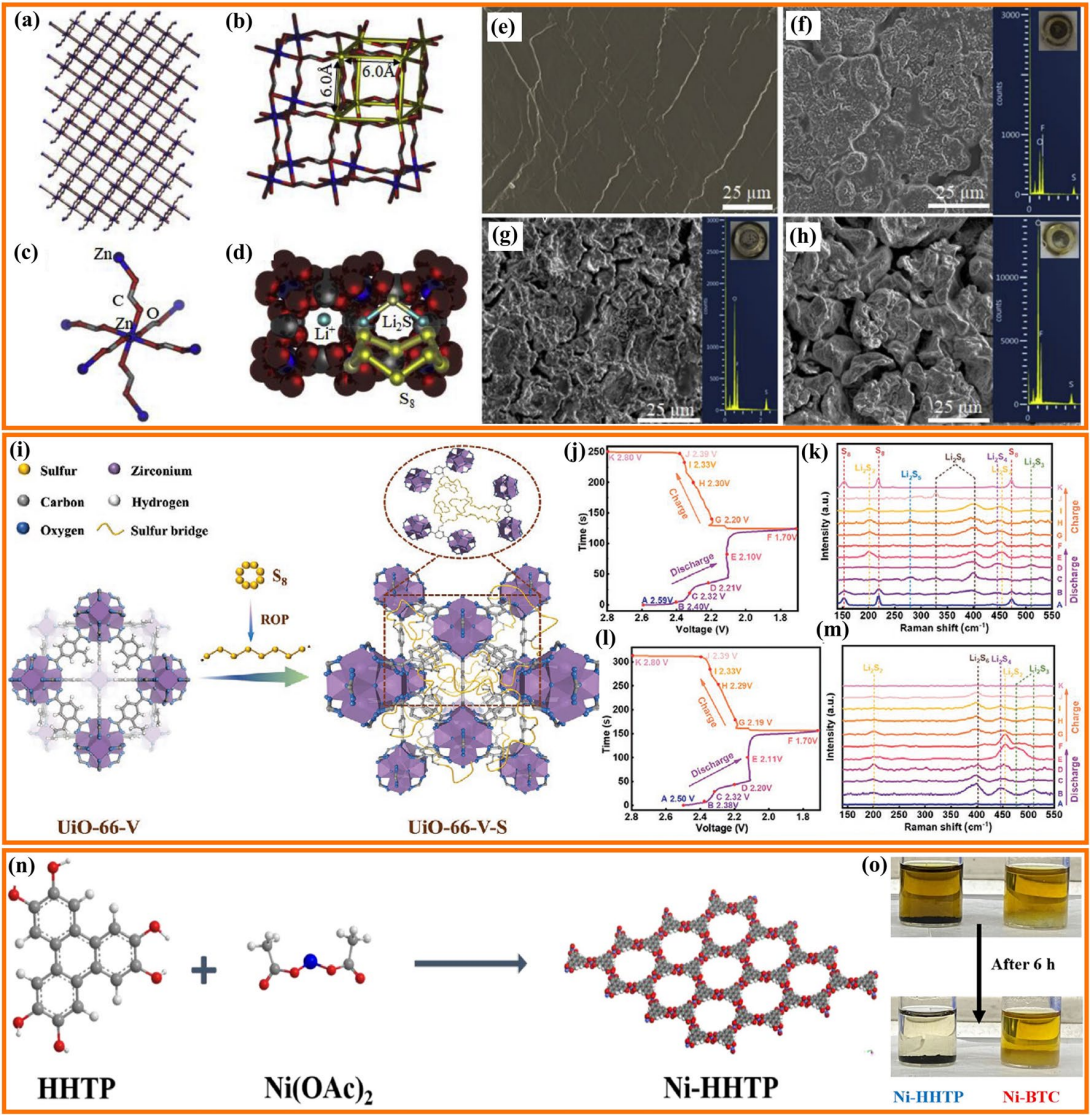
Crystal structure representation of the 3D network DMAZF, where **a** crystal structure packing. **b** Size of the network window into the void space. **c** Metal node displaying the carbon and oxygen coordination to Zn. **d** Description of the LiPS mitigation process and the entry of Li ions through the nanopores. **e** Surface morphology of the uncycled anode of DMAZF/CNTs/sulfur electrode. **f** Cycled anode in the presence of DMAZF/CNTs/sulfur electrode. The surface morphology of the cycled anode **g** Br-UIO-66/CNTs/sulfur electrode and **h** UIO-66/CNTs/sulfur electrode [271]. Copyright 2020, Elsevier. **i** Schematic diagram of the preparation of UiO-66-V-S. **j** Voltage–time curves of UiO-66-V/S cathode. **k** In situ Raman spectra of UiO-66-V/S cathode. **l** Voltage–time curves of UiO-66-V-S cathode. **m** In situ Raman spectra of UiO-66-V-S cathode [258]. Copyright 2022, Wiley-VCH. **n** Schematic illustration of the fabrication of Ni-HHTP. **o** Polysulfides adsorption experiment of Ni-HHTP and Ni-BTC [272]. Copyright 2021, Elsevier

In 2020, Knibbe’s group demonstrated that Lewis acid–base interactions between MOFs and LiPS are beneficial to improving the electrochemical performance of Li–S batteries [271]. In this work, they combined dimethylammonium zinc formate (DMAZF) MOF with CNTs as a molecular sieve to mitigate LiPS migration (Fig. 19a–c). This MOF can act as a molecular sieve that inhibits the migration of LiPS while allowing the diffusion of Li^+^ (Fig. 19d). The authors compared the different morphologies of the Li anode of DMAZF/CNTs/sulfur, Br-UIO-66/CNTs/sulfur and UIO-66/CNTs/sulfur after cycling (Fig. 19e–h). Compared with the Li anodes of Br-UIO-66 (Fig. 19g) and UIO-66 (Fig. 19h), the lithium anode of DMAZF (Fig. 19f) is still relatively smooth. This indicates that DMAZF inhibits the LiPS shuttle better than Br-66 and UIO-66. Owing to this novel nanoporous structure, the DMAZF/CNTs/sulfur composite cathode with a sulfur loading (5 mg cm^−2^) showed a sustainable capacity of 1007 mAh g^−1^ at 0.1C under a low E/S (6 μL mg^−1^).

Besides the interactions between MOFs and LiPS, Huang’s group investigated the effect of sulfur copolymerization with vinyl-functionalized MOF on the performance of Li–S batteries [258]. A MOF-sulfur copolymer (CNT@UiO-66-V-S) was designed as shown in Fig. 19i, in which sulfur chains were copolymerized with vinyl-functionalized MOF (UiO-66-V) at 200 ℃. For comparison, the traditional MOF/S composites (CNT@UiO-66-V/S) were obtained by heat-treating at 155 °C for 12 h. In situ Raman spectroscopy was applied to monitor the real-time transformation processes of LiPS during the first discharge cycle at 0.2C (Fig. 19j-m**)**. A very slow conversion process was observed for the Li–S cells with the traditional UiO-66-V/S electrode (Fig. 19j, k). However, the Li–S cells with the UiO-66-V-S cathode in Fig. 19l, m show a different reaction process. During the discharge process, in situ Raman spectroscopy measurements showed the gradual intercalation process from Li_2_S_8_ to Li_2_S_2_. During charging, all the phase changes are reversible. These results indicate that UiO-66-V-S cathode facilitates the conversion of LiPS to Li_2_S_2_ and reduces the LiPS retention time compared to the traditional conversion pathways in UiO-66-V/S. As a result, such a copolymerization strategy can provide long-term stable cycling performance at a relatively low E/S ratio (10 μL mg^−1^). In addition to conventional insulating MOFs, Wang and his colleagues synthesized conductive MOFs to facilitate the conversion of LiPS [272]. In this work, a conductive Ni-HHTP (HHTP = 2,3,6,7,10,11-hexahydroxytriphenylene) MOF grown on carbon paper is proposed to promote the transformation of LiPS. Ni-HHTP was prepared by the reaction of nickel acetate (Ni(OAc)_2_) and p-conjugated 2,3,6,7,10,11-hexahydroxytriphenyl (HHTP) under solvothermal conditions (Fig. 19n). To show the superiority of conductive Ni-HHTP, the authors used insulating Ni-1,3,5-benzenetricarboxylic acid (Ni-BTC) as a control sample for comparison. Besides the superb electronic conductivity, Ni-HHTP also has strong chemical adsorption to LiPS. As shown in Fig. 19o, Ni-HHTP decolorized the LiPS solution after 6 h, while the LiPS solution with Ni-BTC still had a yellow color. Thanks to the porous structure and high conductivity, the Ni-HHTP@CP cathode exhibited excellent performance at a high sulfur loading (5.4 mg cm^−2^) and decreased electrolyte dosage (E/S = 6.5 μL mg^−1^). Besides the typical MOFs discussed above, our group demonstrated that cationic MOF-based Cu-Mo bimetallic doped carbon nanofibers (Cu-Mo@NPCN) can effectively catalyze the conversion of LiPS. Since the coordination valence state of Cu and Mo improves the electrical conductivity and the catalytic activity, while the intertwined network enhances the Li^+^ diffusion and suppresses the volume change of the electrode, the high sulfur-loaded Li–S cells based on Cu-Mo@NPCN/S cathodes exhibit high areal capacity and good cycling performance, suggesting its great potential for practical applications.

MOFs have coordinated metal ions and porous structures; they can strongly bind to LiPS. However, most of the MOFs have low electronic conductivity, which results in low utilization of sulfur species. In addition, the presence of open metal centers as Lewis acid sites and the pore size tuning of MOFs to optimize sulfur utilization are the focus of current research, but the interaction mechanism between MOFs and LiPS needs to be investigated in more detail.

### Single Atoms

Recent years have seen the development of numerous single-atoms (SAs) catalysts (Fe, Co, Ni, Mo, W, Cu, etc.) that exhibit superior activity for a variety of chemical reactions [106, 136, 277, 283–285]. These catalysts have become available through a variety of cutting-edge atomic-scale techniques, including synthetic methods, theoretical calculations and characterization tools. SA catalysts maximize the dispersion of the active metal phase. Besides maximizing metal utilization, SA catalysts with uncoordinated bonds provide strong chemisorption and excellent catalytic activity in Li–S batteries, promoting LiPS conversion and well-controlled Li_2_S deposition sites with low E/S ratio [142, 276, 286]. Table 8 shows a summary of SAs catalyst and their properties for the performance of lean electrolyte Li–S batteries.

**Fig. 20:**
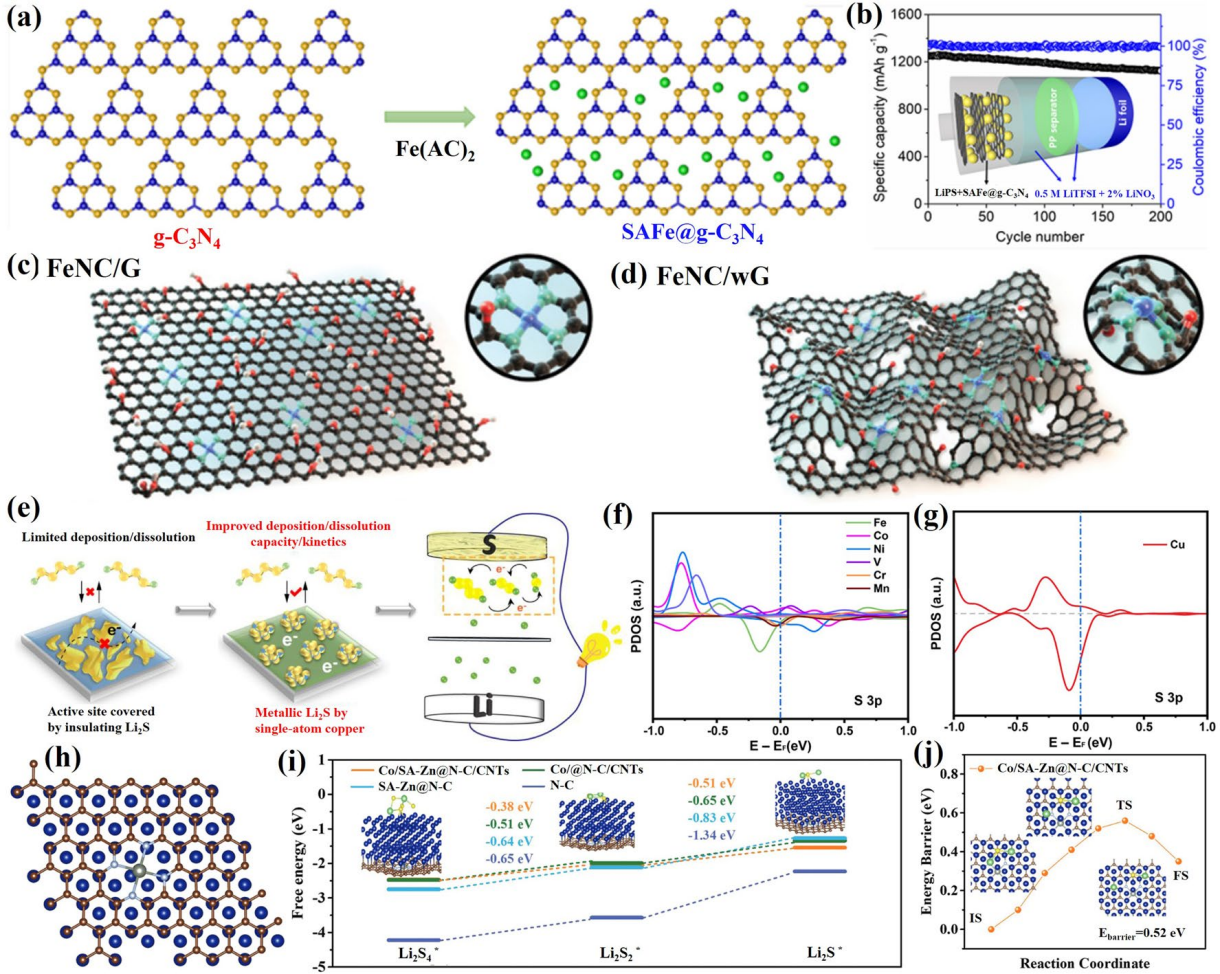
**a** Synthesis process of SAFe@g-C_3_N_4_. **b** The corresponding electrochemical performance of SAFe@g-C_3_N_4_-based Li–S cell [283]. Copyright 2020, American Chemical Society. Schematic diagram of FeNx sites on **c** planar graphene (FeNC/G) and **d** wG (FeNC/wG [101]. Copyright 2022, Wiley-VCH. **e** Schematic representation of Li_2_S precipitation/decomposition on SA-Cu@NCNF. PDOS of S 2*p* in **f** SATM@NG/Li_2_S (TM = Fe, Co, Ni, V, Cr, Mn) and **g** SA-Cu@NG/Li2S [275]. Copyright 2022, Elsevier. **h** Top view of the optimized configurations of Co/SA-Zn@N-C/CNTs. **i** Energy profiles for LiPS reduction on Co/SA-Zn@N-C/CNTs, Co@N-C/CNTs, SA-Zn@N-C and N-C. **j** Energy profile of Li_2_S delithiation on Co/SA-Zn@N-C/CNTs [274]. Copyright 2022, Wiley-VCH

Han et al. applied DFT calculations to explore the catalytic activity of nine types of 3d SAs metal catalysts (Cu, Ni, Co, Fe, Mn, Cr, V, Ti, Sc) [287]. By analyzing the d-p orbital hybridization between metal atoms and S species, they discovered that single-atom-Ti (SATi) exhibits strong d-p hybridization and forms more robust bonds with LiPS, as Ti has slightly filled anti-bonding states. The more efficient d-p orbital hybridization in the SATi-S bond lowers the energy barriers of LiPS reduction/Li_2_S oxidation. As a result, the Li–S cells based on SATi as sulfur hosts achieved a high electrochemical specific capacity. However, these electrochemical results were evaluated under flood electrolyte conditions. Chen’s group prepared highly active SA iron@graphitic carbon nitride catalytic materials (SAFe@g-C_3_N_4_) to find the balance between low E/S ratio and high electrochemical performance (Fig. 20a) [283]. The atomic ratio of N and Fe elements in SAFe@g-C_3_N_4_ was estimated to be 54.17 and 0.73%. The nitrogen sites in the g-C_3_N_4_ structure allow for more SAs loading and suppress the shuttle effect of LiPS during the charge–discharge process. The Li–S cells based on SAFe@g-C_3_N_4_ show superior cycling stability and maintain a capacity of 90% after 200 cycles at 0.2C. Other SAs catalysts also show outstanding performance at low E/S ratio. For example, Wang et al. reported a novel template-free folic acid self-assembly strategy for the simple preparation of ultrathin N-doped carbon nanosheets confined to single-metal-atom catalysts [273]. Co–N–C single-metal-catalysts as sulfur hosts could achieve long-term stability with a low E/S ratio of 4 µL mg^−1^and a high sulfur loading of 6 mg cm^−2^.

Morphology also plays an important role in the electrochemical properties of SAs. Kim et al. described the morphological engineering of a graphene support to modify the local coordination structure of FeN_4_ moieties (Fig. 20c, d) [101]. Compared to FeN_4_ sites on planar graphene substrates (G), FeN_4_ active sites supported on a highly wrinkled graphene (wG) matrix (FeNC/wG) induced the modification of the local structure of Fe, thereby promoting the conversion reaction kinetics of LiPS. The relative content of Fe in FeNC/wG was estimated to be 0.78 wt%. Due to the beneficial interaction between the atomically modified FeN_4_ active sites and the morphological engineering of graphene support, the Fe–N–C-based Li–S batteries showed excellent cycling stability with low electrolyte usage. The morphology of Li_2_S deposition has a significant impact on the deposition capacity of Li_2_S. Li’s group proposed that nitrogen-doped carbon fibers decorated with SA Cu change the Li_2_S deposition morphology from the traditional 2D lateral morphology to 3D spherical clusters by consecutive 3D nucleation and growth (Fig. 20e) [275]. The atomic ratios of N and Cu were around 3.03 and 0.25%, respectively. The authors first performed DFT calculations to fully explore the electronic characteristics of Li_2_S on several SAs catalysts with TM-N_4_@C structure. Li_2_S exhibits a half-metallicity (metallicity in one electron spin direction) on SA-Fe, Co, Ni, V, Cr and Mn (Fig. 20f). However, Li_2_S adsorbed on SA-Cu is two-electron spin orientated and shows metallicity, with the highest electron density of states (DOS) around the Fermi energy level (E_F_), thus significantly increasing the electronic conductivity of the Li_2_S molecule (Fig. 20g). As a result, the precipitation capacity of Li_2_S and the effectiveness of Li_2_S-covered catalytic sites are enhanced. Most current work for SAs systems has focused on generating high densities of individual single metal atoms and avoiding the formation of metal-containing nanoparticles. Wang et al. embedded both Co nanoparticles and single-atom Zn into nitrogen-doped porous carbon nanosheet-grafted CNTs (denoted as Co/SA-Zn@N–C/CNTs) as electrocatalysts for Li–S batteries [274]. The Co and Zn contents in the Co/SA-Zn@N–C were around 4.32 and 0.94 wt%, respectively. In addition, N-doped carbon nanosheets (N–C), N-doped carbon nanosheets embedded with Zn single atoms (SA-Zn@N–C), and N-doped carbon/CNTs embedded with Co particles (Co@N–C/CNTs) were also prepared for comparison. DFT calculations were performed to gain insight into the catalytic mechanism of the LiPS conversion with Co/SA-Zn@N–C/CNTs. Figure 20h shows the optimized configuration of the Co/SA-Zn@N–C/CNTs. Figure 20i presents the Gibbs free energy diagrams for the conversion reactions on these four materials. The lowest Gibbs free energy for the rate-limiting step is found in the integrated Co/SA-Zn@N–C/CNTs, indicating that the co-existence of single Zn atoms and Co nanoparticles significantly lowers the energy barrier for the solid–solid Li_2_S_2_-Li_2_S conversion reaction. The authors also investigated the dissociation ability of Li_2_S on the four configurations during the charging process (Fig. 20j). The energy profiles of Li_2_S dissociation also showed that the energy barrier for decomposition was lower on Co/SA-Zn@N–C/CNTs (0.52 eV) than on N–C (1.61 eV), SA-Zn@N–C (1.31 eV) and Co@N–C/CNTs (0.77 eV), indicating that the SA-Zn sites and Co sites could co-operate to speed up the delithiation of Li_2_S. This work proposed coupled Co nanoparticles and SA-Zn moieties with an optimal charge redistribution to catalyze the LiPS conversion reactions.

Overall, SAs catalysts hold great potential for use in Li–S batteries due to their high atom utilization efficiency, strong catalytic activity and the ability to adjust their structure at the atomic level. Although SAs catalysts offer the above-mentioned advantages, the development of large-scale synthesis strategies which allow increased loadings is crucial for their application in lean electrolyte Li–S batteries.

### Other Transition Metal Compounds

In addition to the previously mentioned transition metal-based systems, the applications of transition metal borides, transition metal fluorides and nickel foam in lean electrolyte Li–S batteries are also discussed in this section. Table 8 summarizes recent investigations on these compounds and their corresponding energy landscape in Li–S batteries.

**Fig. 21:**
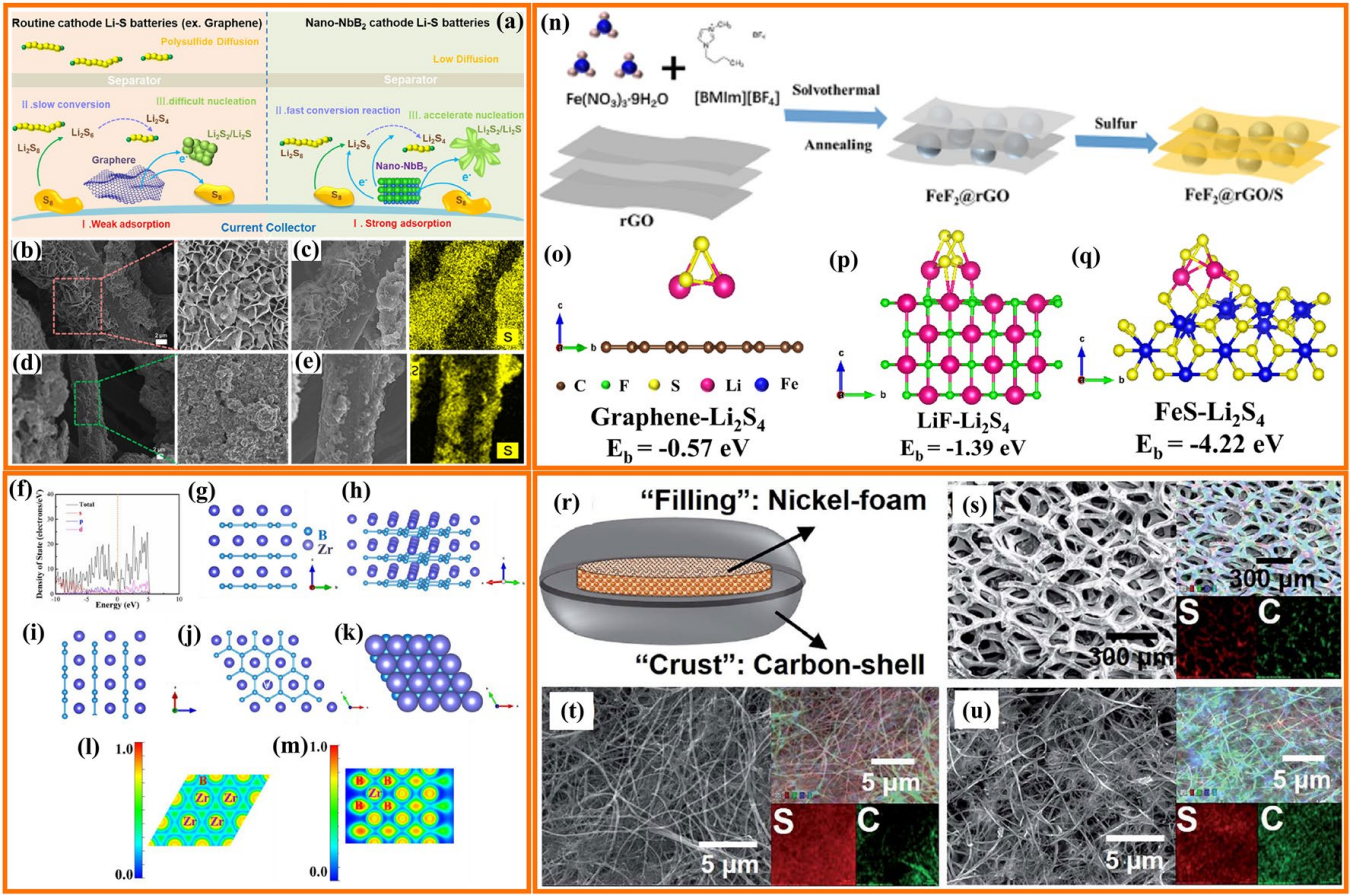
**a** Illustration of NbB_2_ promoting LiPS transformation and facilitating Li_2_S deposition. SEM images of Li_2_S precipitation: **b** NbB_2_ and d NPG. Corresponding EDS mapping of S element: **c** NbB_2_ and **e** NPG [278]. Copyright 2022, American Chemical Society. **f** Partial density of states of ZrB_2_. **g**–**j** Cell structure of hexagonal ZrB_2_. **k** Surface schematic of ZrB_2_ (001). Electron location function (ELF) of ZrB_2_: **l** Zr and B atoms at (001) surface, **m** Zr and B atoms at (100) surface [279]. Copyright 2022, Elsevier. **n** FeF_2_@rGO Synthetic route diagram. Atomic configuration and corresponding binding energy after adsorption of Li_2_S_4_, **o** rGO, **p** LiF and **q** FeS [281]. Copyright 2022, Elsevier. **r** Illustration of a nickel-foam@carbon-shell cathode with pie-like architecture. SEM/EDX examination of s nickel-foam, t inner and u outer sides of the carbon shell removed from a cycled nickel-foam@carbon-shell cathode [282]. Copyright, Royal Society of Chemistry

Transition metal borides have recently been applied as sulfur hosts for lean electrolyte Li–S batteries due to their high metallic conductivity [288–290]. Unlike other polar metal compounds, boron (B) has an electronic structure with empty orbitals that can trap LiPS by forming B-S bonds. Hence, both the metal and B can chemisorb LiPS to suppress the shuttle effect [280, 288, 291]. In 2018, Xu’s group first reported the use of titanium diboride (TiB_2_) as a sulfur host [292]. In 2022, Xu’s group designed niobium diboride (NbB_2_) nanoparticles with high conductivity and catalytic activity toward LiPS conversion for Li–S cells with low-electrolyte content [278]. Figure 21a shows the mechanism of NbB_2_ nanoparticles facilitating LiPS transformation and Li_2_S 3D-nucleation: (1) NbB_2_ nanoparticles with high electrical conductivity and a high number of catalytic active sites facilitate the reaction kinetics of LiPS; (2) nano-sized NbB_2_ exposes more catalytic sites for the conversion of LiPS; (3) both metal Nb and B can chemisorb LiPS and direct Li_2_S nucleation. Nitrogen-phosphorus co-doped graphene (NPG) composites were utilized as comparison samples. The authors used SEM to illustrate the effect of different host interfaces on the precipitation morphology of Li_2_S to reveal the nucleation mechanism of Li_2_S. As shown in Fig. 21b, d, Li_2_S particles aggregated on the surface of the reference NPG. However, 3D “flower-like” Li_2_S flakes are deposited on the surface of NbB_2_, suggesting fast and uniform nucleation of Li_2_S on the surface of NbB_2_. In addition, EDS mapping also demonstrated that the coverage of Li_2_S on the NbB_2_ surface exceeds that of NPG (Fig. 21c, e). As a result, the NbB_2_/NPG/S composite showed an initial capacity of 1030 mAh g^−1^ and maintained 647 mAh g^−1^ after 40 cycles at 0.1C with a sulfur loading (16.5 mg cm^−2^) and a low E/S ratio (6 μL mg^−1^). Almost simultaneously, their group also proposed highly conductive zirconium boride (ZrB_2_) nanoparticles as bidirectional catalysts for lean electrolyte Li–S batteries [279]. As shown in Fig. 21f, the partial density of states indicates that ZrB_2_ is continuous at the Fermi level, proving its metallic nature. ZrB_2_ is a hexagonal crystal system (Fig. 21g-j). The graphite-like structure of the B atomic layer structure and the outer electronic structure of Zr determine that ZrB_2_ has good conductivity. Furthermore, the surface unsaturated coordination number of Zr atoms (Fig. 21k) leads to a high affinity of ZrB_2_ for LiPS. In addition, the electron location function (ELF) indicates that the Zr atom has good metal properties (Fig. 21l), and the B atom has perfect electron localization (Fig. 21m). These indicate that ZrB_2_ can be used as sulfur hosts for Li–S batteries. However, it is difficult to fabricate pure phase ZrB_2_ because it usually requires high temperatures. Here, the authors prepared ultrafine ZrB_2_ nanoparticles by a solid-state synthesis at a relatively low temperature. The obtained ZrB_2_/nitrogen-doped graphene/S (defined as “ZrB_2_/NG/S”) cathode delivers a low-capacity loss of 0.219% per cycle at 0.1C with a sulfur loading (4.8 mg cm^−2^) and low-electrolyte content (E/S ratio = 8 μL mg^−1^).

Besides transition metal borides, transition metal fluorides have recently been utilized as electrocatalysts for high-sulfur loading with lean electrolyte Li–S cells. For example, Zhang’s group synthesized FeF_2_@rGO composite via a solvothermal method (Fig. 21n) [281]. They found that FeF_2_ converts in situ into LiF and FeS during the discharge process. LiF with a low barrier for Li^+^ ion diffusion can enable fast Li^+^ ion diffusion. Also, FeS exhibited high affinity to LiPS and catalytic activity for LiPS conversion. DFT calculations analyzed the binding energy between Li_2_S_4_ and the different cathode components after in situ transformation. As shown in Fig. 21o-q, FeS has the most robust adsorption capacity on LiPS with a binding energy of − 4.22 eV. As a result, even under high sulfur loading (12.7 mg cm^−2^) and lean electrolyte conditions (6 μL mg^−1^), the cells with FeF_2_@rGO as cathode exhibit a high areal capacity of 12.3 mAh cm^−2^.

During the past decade, the use of nickel foam as an electrode substrate in Li–S cells has been investigated. For example, Manthiram’s group proposed a new pie-like electrode consisting of an electrocatalytic nickel foam and an outer carbon shell for high-energy Li–S batteries with low-electrolyte content (Fig. 21r) [282]. The nickel foam features a 3D porous structure and an uneven surface to achieve high sulfur loading and rich active sites. Meanwhile, the carbon shell consists of an interwoven network of carbon nanotubes and carbon nanofibers, which can retain polysulfides. Figure 21s-u shows the SEM images of the nickel foam and carbon shell after 100 cycles. There is no significant change in their morphology, which means they have excellent mechanical strength. This pie-like structure allows the cathode to have an ultrahigh sulfur loading of 40 mg cm^−2^ and achieve a high areal capacity of over 40 mAh cm^−2^ at 0.2C with a low E/S ratio of 7. This work provides a new strategy for developing optimized cathodes to achieve high energy density Li–S batteries.

## Conclusions and Perspectives

This review provided a systematic summary of a range of composites of carbon with transition metal-based compounds used in lean electrolyte Li–S batteries. The first topic of discussion was the fundamentals and limitations of the electrochemical conversion of LiPS with low E/S ratios. Then, the functional mechanism and characteristics of the transition metal-based carbon compounds for lean-electrolyte Li–S cell cathode components were comprehensively reviewed. Finally, several possible improvement trends for Li–S batteries in low electrolyte environments were foreseen. Specifically, we discussed the application of carbon-based sulfur hosts containing metal elemental, alloy nanoparticles, metal oxides, phosphides, chalcogenides, nitrides, carbides, borides, fluorides, as well as metal organic frameworks and single atoms of these compounds in lean electrolyte Li–S batteries. By exploiting the inherent characteristics of hybrid nanostructure geometries and developing their external properties, transition metal-based carbon compound engineering holds a lot of potential and provides a path for constructing high-performance Li–S systems with low electrolyte usage.

While a lot of progress has been made in the development of lean electrolyte Li–S batteries based on these cutting-edge cathode materials, the following points have not received sufficient attention:

i. Heterogeneous electrocatalysts have a limited number of specific catalytic sites; therefore, it is necessary to increase the number of redox-active sites per unit surface area to enhance catalytic activity without changing the catalyst structures. However, the understanding of how the number of active sites influences the electrochemical performance of Li–S cells is still unclear. Therefore, an in-depth study of the relationship between the catalytic site density of catalysts and electrochemical performance of Li–S batteries is required. Furthermore, the catalytic effect of catalysts can be enhanced by increasing the catalyst dosage; however, a large dosage of catalysts can greatly decrease the gravimetric energy density of Li–S cells; thus, a balance needs to be discovered between the high catalyst dosage and the high gravimetric energy density of Li–S cells.
ii. As the redox reaction proceeds, “dead sulfur” can lead to catalyst deactivation; therefore, the effectiveness protection of active sites to maintain sustainable electrocatalytic effects for long-cycle Li–S batteries deserves more attention. Emphasis should be devoted to developing catalysts with improved poisoning resistance to mitigate the negative impacts of "dead sulfur" deposition and catalytic interface poisoning. Furthermore, the exploration of liquid metal catalysts with self-healing and reversible properties holds promise for enhancing the long-term stability and durability of the catalytic system.
iii. The precipitation process and the deposition morphology of Li_2_S on the electrolyte/electrode surface should be studied, as it significantly influences the transport of Li ions and electrons at the reactive interfaces. Optimized reaction interfaces help to regulate the redox conversion of LiPS, control the nucleation of Li_2_S and achieve 3D growth of Li_2_S. Exploring candidates such as stepwise and selective electrocatalysis can offer opportunities for manipulating the kinetics of Li_2_S nucleation versus growth. In addition, the decomposition of Li_2_S also deserves special mention. Li_2_S is hard to be completely oxidized due to the high energy barrier (*E*_b_) of Li_2_S delithiation during charging. In this sense, the design of bidirectional electrocatalysts holds great prospects in accelerating the transformation from insoluble Li_2_S to long-chain LiPS.
iv. The balance of carbon content and electrolyte/sulfur ratio should be carefully considered. The minimization of carbon content in the cathode is very important at low E/S ratios as carbon can lead to severe electrolyte depletion due to high electrode porosity. However, recent studies have shown that the rate constant of sulfur redox in the presence of carbon is significantly increased, indicating the importance of electrical conductivity in accelerating sulfur redox reactions. Thus, the balance between carbon content and electrolyte/sulfur ratio should be studied.
v. More research efforts should be directed toward evaluating the electrochemical performance of Li–S pouch cells, especially at high areal sulfur loadings and low E/S ratios, to achieve high energy density Li–S pouch cells exceeding 400 Wh kg^−1^. The characteristics of multilayer pouch cells and transparent calculations of their components need to be explored further, as these aspects are critical for the commercial application of Li–S batteries.
